# Compliant Iontronic Triboelectric Gels with Phase-Locked Structure Enabled by Competitive Hydrogen Bonding

**DOI:** 10.1007/s40820-024-01387-4

**Published:** 2024-04-09

**Authors:** Guoli Du, Yuzheng Shao, Bin Luo, Tao Liu, Jiamin Zhao, Ying Qin, Jinlong Wang, Song Zhang, Mingchao Chi, Cong Gao, Yanhua Liu, Chenchen Cai, Shuangfei Wang, Shuangxi Nie

**Affiliations:** School of Light Industry and Food Engineering, Guangxi University, Nanning, 530004 People’s Republic of China

**Keywords:** Triboelectric nanogenerator, Cellulose, Triboelectric gel, Self-powered sensor, Energy harvesting

## Abstract

Rapid advancements in flexible electronics technology propel soft tactile sensing devices toward high-level biointegration, even attaining tactile perception capabilities surpassing human skin. However, the inherent mechanical mismatch resulting from deficient biomimetic mechanical properties of sensing materials poses a challenge to the application of wearable tactile sensing devices in human–machine interaction. Inspired by the innate biphasic structure of human subcutaneous tissue, this study discloses a skin-compliant wearable iontronic triboelectric gel via phase separation induced by competitive hydrogen bonding. Solvent-nonsolvent interactions are used to construct competitive hydrogen bonding systems to trigger phase separation, and the resulting soft-hard alternating phase-locked structure confers the iontronic triboelectric gel with Young's modulus (6.8–281.9 kPa) and high tensile properties (880%) compatible with human skin. The abundance of reactive hydroxyl groups gives the gel excellent tribopositive and self-adhesive properties (peel strength > 70 N m^−1^). The self-powered tactile sensing skin based on this gel maintains favorable interface and mechanical stability with the working object, which greatly ensures the high fidelity and reliability of soft tactile sensing signals. This strategy, enabling skin-compliant design and broad dynamic tunability of the mechanical properties of sensing materials, presents a universal platform for broad applications from soft robots to wearable electronics.

**Supplementary Information:**

The online version contains supplementary material available at 10.1007/s40820-024-01387-4.

## Highlights

- A bionic phase-locked structure-inspired iontronic triboelectric gel is proposed with good mechanical compliance for wearable haptic sensing applications.
- Competitive hydrogen bonding systems are constructed through polymer-solvent-nonsolvent interactions, and regeneration of polymers with weak hydrogen bond donors triggers controlled phase separation.
- Self-powered haptic skin constructed with iontronic triboelectric gel has a modulus (150.6 kPa) and stretchability (> 400%) similar to that of the human body, enabling fidelity transmission of haptic signals and precise recognition of sensing objects.

**Supplementary Information:**

The online version contains supplementary material available at 10.1007/s40820-024-01387-4.

## Graphical abstract

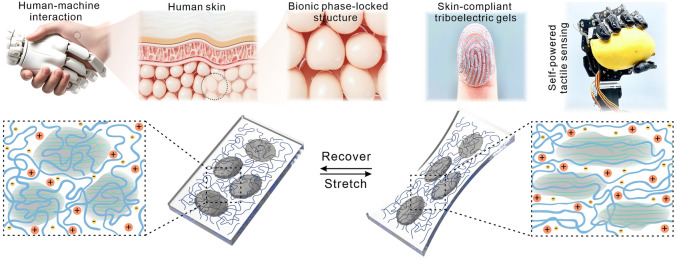

## Introduction

Demonstrating the potential of flexible electronics technology to merge the realms of information and biology, wearable tactile sensing devices have attracted considerable attention within the domain of human–machine interaction systems [1–3]. Triboelectric sensing devices, relying on contact electrification and electrostatic induction coupling effects, possess the capability to transform applied mechanical stimuli into electrical signals, thereby streamlining the acquisition and quantification of tactile information [4–6]. The user-friendly self-powered sensing mechanism facilitates wireless tactile perception and material identification, progressively gaining preference in emerging electronic products, including soft robots, artificial prosthetic skins, and deep space probes [7–9]. Large-scale commercial elastomers (polydimethylsiloxane, thermoplastic polyurethanes, silicone rubber, etc.) have long been widely used as stretchable, elastic triboelectric materials due to their ease of access and excellent reprocessing properties [10]. However, Young's modulus of these materials is often too high, making it challenging to simulate the realistic touch of human skin in practical applications [11, 12]. The high mismatch of modulus and extremely low adhesion make them difficult to form compliant conformal contacts and stable interfacial connections with human bodies and robots. This impairs the operational stability of wearable tactile sensing devices as well as the fidelity transmission of sensing signals, and severely limits their functional expansion in human–machine interaction [13, 14]. In light of these challenges, there is an urgent need to propose a design strategy for compliant elastic triboelectric materials to optimize the mechanical suitability of wearable tactile sensing devices [15–17].

In the development of sensing materials (e.g., hydrogels, ionogels, or elastomers) for wearable soft tactile sensing devices, the challenge of harmonizing modulus, elasticity, and strength has emerged as a pivotal obstacle that hinders their progress [18–20]. Most construction methodologies, such as polymer blending, small molecule doping, and graft copolymerization, often yield materials with a modulus akin to that of skin but at the cost of sacrificing elasticity and strength (typically falling below 100 kPa). This trade-off diminishes the practical functionality of these materials [21]. In materials chemistry, phase-locked structures constructed via phase separation are regarded as an effective strategy for regulating the mechanical properties of gel materials and have emerged as promising candidates for solving the above problems [22–24]. Phase separation arises from differences in polymer solubility, resulting in the spontaneous formation of two-phase structures, where the hard phase is embedded in the soft phase to form a self-assembled phase-locked structure [25]. In this structure, rigid, hard phases dissipate a substantial amount of energy during stress, while flexible, soft phases prevent stress concentration and enable substantial deformation [26, 27]. Block copolymerization of polymers is the most common method for inducing phase separation, where soft and hard chain segments are constructed by controlling the content of each component [28]. However, giant stiffness switching and harsh conditions (usually requiring external stimuli, e.g., humidity, temperature, and stress) limit its application prospects in soft electronic devices [29–31]. In this context, elucidating the formation mechanism of soft-hard alternating phase-locked structures and regulating the interactions during phase separation is particularly important for the advanced fabrication of soft tactile sensing materials.

The human subcutaneous tissue consists of stearin particles embedded in a viscoelastic matrix that share the flexibility and strength of the skin and exhibit excellent mechanical compliance [32]. Inspired by this natural biomimetic structure, this study reports a mechanically compliant iontronic triboelectric gel with phase-locked structure, which arises from phase separation induced by competitive regeneration of hydrogen bonds between polymer networks. Solvent-nonsolvent interactions are used to construct competitive hydrogen bonding systems. Strong hydrogen bond donors compete for anions and form a soft phase to dissipate stress, while weak hydrogen bond donors lose anions and aggregate, regenerating into a hard phase to dissipate energy. Benefiting from an effective interphase load transfer mechanism in phase-locked structure, the iontronic triboelectric gels maintained Young's modulus in the range matching human skin (6.8–281.9 kPa) while possessing high tensile strength (> 300 kPa), high elongation (880%), and high toughness (1.15 MJ m^−3^). Notably, the excellent bionic mechanical properties enable the iontronic triboelectric gel to achieve compliant bonding with human and robotic surfaces. It also achieves conformal contact with sensing objects, realizing precise feedback of wearable tactile sensing signals and self-powered recognition of objects made of different materials. This study provides a convenient solution for the development of elastic triboelectric materials with compliant mechanical properties, which is expected to promote the further application of soft electronics in tactile sensing.

## Experimental Section

### Materials

2-Hydroxyethyl methacrylate (HEMA), poly(ethylene glycol) dimethacrylate (PEGDA), Irgacure 184, α-cellulose, chitosan, and soluble starch were purchased from Sigma-Aldrich. Before use, a chromatographic column was utilized to remove the blocker from the HEMA and the remaining reagents did not require further purification. Silk fibroin protein was purchased from Guanying Biotechnology Co., LTD. 1-butyl-3-methylimidazolium chloride ([Bmim]Cl) was purchased from Lanzhou Institute of Chemical Physics. A vacuum oven was utilized to remove moisture before use. Ecoflex 0030 was purchased from Smooth-On. Polydimethylsiloxane (PDMS) was purchased from Dow Corning. The conductive silver paste was purchased from Shenzhen Xinwei New Material Co., LTD. Calcofluor White Stain was purchased from Shanghai Maokang Biotechnology Co., LTD.

### Preparation of Triboelectric Gels

Before constructing the samples, cellulose and [Bmim]Cl were treated in a vacuum oven at 90 °C for 48 h to remove water. A viscous and homogeneous system of cellulose/[Bmim]Cl (cellulose content ranging from 0 to 2.5 wt%) was prepared by thermal dissolution at 90 °C for 8 h. HEMA, Polyethylene glycol dimethacrylate (PEGDA), and Irgacure 184 were homogeneously mixed into a clear precursor solution and slowly added to the cellulose/[Bmim]Cl system. where the molar ratio of HEMA to [Bmim]Cl was 1:1. The Irgacure 184 and the crosslinker PEGDA were both set to 0.1 mol% (relative to the amount of monomer). The well-mixed solution was then degassed, injected into silica gel molds, and finally cured by UV light (365 nm). Chitosan, silk protein, and soluble starch are used in the same way as cellulose, with solubility controlled only by adjusting the heating temperature.

### Characterization

ATR-FTIR (Nicolet iS 10, Thermo Fisher Scientific, USA) was used for characteristic peak measurements with a resolution of 0.4 cm^−1^ and a wavelength range of 4000–400 cm^−1^. The crystal structure of RCPTG was analyzed by X-ray diffraction (XRD) using Cu Kα radiation (MiniFlex600). Wide-angle X-ray scattering (WAXS) (Nanostar, Bruker, Germany) was used to observe the phase inside the RCPTG with scattering vectors in the range of 0.2–2.5 Å. Phase separation within RCPTG was observed using confocal laser scanning microscope (CLSM) (FV3000, Olympus, Japan) and fluorescence microscope (DM4B, Leica, Germany), and the regenerated cellulose was stained using Calcofluewhite Stain before observation. A UV spectrophotometer (UV-3600Plus, Shimadzu, Japan) was used to detect the transmittance of RCPTG in the visible range of 380–780 nm. The surface texture of RCPTG was photographed by a super depth-of-field microscope (VHX-6000, KEYENCE, Japan). The soft-hard alternating phase-locked structure inside the RCPTG was visualized by nano-CT (SKYSCAN 2214, Bruker, Germany) for X-ray tomography. The atomic force microscopy (AFM) phase distribution of the RCPTG on microsubstrates were measured by tapping-mode AFM (Dimension Icon, Bruker, Germany) using tapping MPP-rotated cantilevers with silicon probes. Chemical bond analysis was performed using K-Alpha X-ray photoelectron spectroscopy (XPS), and with test voltage, current, and incident angle set to 12 kV, 16–25 mA and 90°, respectively (ESCALAB 250XI + , Thermo Fisher Scientific, USA).

### Mechanical and Rheological Testing

Tensile, compression, and peel tests were performed using an electronic universal testing machine (3367, Instron, USA). The tensile samples were dumbbell-shaped with a tensile size of 20 × 4 × 2 mm^3^ and a tensile speed of 50 mm min^−1^. The compression samples were cylindrical with a diameter of 30 mm. The peeling samples were strips with a size of 20 × 100 × 1 mm^3^, which were pressed against the substrate material for 1 min using a heavyweight before the test, and the peeling speed was 100 mm min^−1^. Dynamic viscoelasticity tests were performed on RCPTG using a rheometer (HR 20, TA Instruments, USA), including linear viscoelastic region (LVR) tests, angular frequency tests, and variable temperature tests.

### Thermodynamic Analysis and Environmental Stability

A simultaneous thermal analyzer (STA 449F5, NETZSCH, Germany) was used to compare the thermodynamic stability of Cellulose-ionogel, and RCPTG over the range of 25–600 °C. After successful RCPTG preparation, the samples were exposed to the laboratory environment as well as placed in a vacuum oven to check their hygroscopic properties and water content, respectively. The leakage of ionic liquid was checked by wrapping the RCPTG with dry dust-free tissue paper.

### Cytotoxicity and Antimicrobial Properties Testing

To evaluate the biocompatibility of RCPTG, the cytotoxicity of RCPTG on NIH3T3 (mouse embryonic fibroblast) was tested using Live-dead staining. RCPTG with a size of 10 × 10 × 1 mm^3^ was immersed in a cell culture medium of 48-well plates with NIH3T3 for 48 h. Calcein-AM was utilized as a fluorescent probe to stain the live cells and CLSM was used for observation. The UV-sterilized RCPTG was co-cultured with Escherichia coli and Staphylococcus aureus to check the antimicrobial properties. Breathable dressings with RCPTG were adhered to the small arms of human research participants for 48 h to check skin sensitivity.

### Electrical and Sensing Testing

A laser engraver (VLS3.50-SYS, Universal, USA) was utilized to etch flexible circuit shapes on a polyethylene film, which was used as a mask to cover the RCPTG. Conductive silver paste was applied on top of the mask and removed after drying for 24 h. The dried flexible circuits acted as the back electrode of a single-electrode triboelectric nanogenerator for inducing electric charge. Several 2 × 2 cm^2^ RCPTG-skins were assembled into single-electrode triboelectric nanogenerator arrays by assembling them on a manipulator. Triboelectric signals from the RCPTG-skins were taken using a linear motor (LinMot E1100, Switzerland), an electrostatic meter (Keithley 6514, USA), and an acquisition card (NI-USB6259, USA). A 1.58 t vehicle was driven over the RCPTG-skin to verify its mechanical robustness and sensing stability. The upper and lower white plastic plates are used to prevent RCPTG from adhering to the tires.

## Results and Discussion

### Design Principle of Bioinspired Compliant Iontronic Triboelectric Gels

**Fig. 1:**
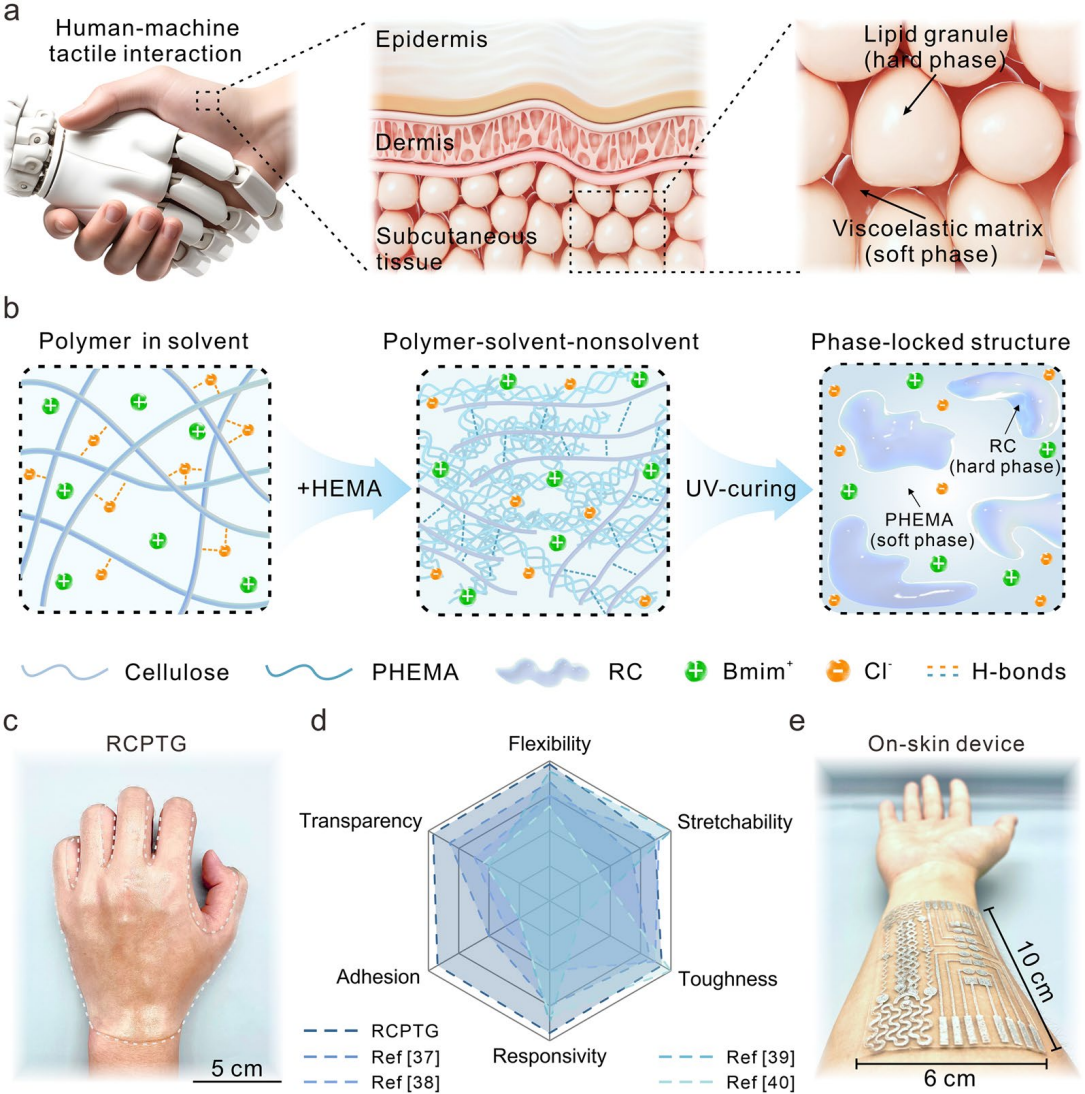
Design principle of bioinspired compliant triboelectric gels. **a** Natural phase-locked structures in human subcutaneous tissue. **b** Process of inducing phase separation via solvent-unsolvent effects to construct phase-locked structures. **c** Compliant contact of RCPTG with human skin without any adhesive. **d** Properties of RCPTG compared to reported elastic triboelectric materials, including flexibility, stretchability, toughness, triboelectric responsivity, adhesion, and transparency. **e** RCPTG-based on-skin device is loaded on the small arm of the human body

In human subcutaneous tissue, discontinuous and dispersed lipid granules (hard phase) are encapsulated in a continuous viscoelastic matrix (soft phase). The former is used as an energy dissipation domain during skin stretching, and the latter is subjected to greater strain than the dermis, together contributing to the low modulus and high elasticity of the entire subcutaneous tissue (Fig. 1a) [33]. This effective interphase load transfer mechanism in natural phase-locked structures inspires the mechanomimetic design of elastic triboelectric material. To construct a controlled phase separation, hydroxyethyl methacrylate (HEMA) was introduced into a homogeneous0 cellulose/ionic liquid solution to form a polymer–solvent-nonsolvent ternary system (Fig. 1b). The degree of phase separation is closely related to the ratio of the three components. Here, the phase separation is controlled by modulating the cellulose content. The ionic liquid 1-butyl-3-methylimidazolium chloride ([Bmim]Cl) is a good solvent for cellulose. During dissolution, the smaller anions act as hydrogen bond acceptors to form strong hydrogen bonds with hydroxyl protons on cellulose to dissociate the fiber bundles; the larger cations are dispersed in the middle of the chain to act as barriers [34]. HEMA has little or no solvency for cellulose, however, it has much stronger hydrogen bond donor and is able to preferentially hydrogen-bond with the anions. Competitive hydrogen bonding systems were constructed by taking advantage of the different affinities of cellulose and HEMA for anions. Strong hydrogen bond donors in HEMA competitively compete for anions; the originally dissolved cellulosic molecular chains re-form interchain hydrogen bonds due to the loss of anions and aggregate as regenerated cellulose (RC). The RC forms the rigid phase and the poly(hydroxyethyl methacrylate) (PHEMA) forms the soft phase, resulting in the formation of Regenerated Cellulose/PHEMA triboelectric gels (RCPTGs) with soft-rigid alternating phase-locked structure.

The rigid RC network in the rigid phase provides RCPTGs with considerable strength; the highly flexible PHEMA network maintains the modulus of the gel in a range similar to that of human skin [35]. In addition, the abundance of hydroxyl groups in PHEMA enables RCPTG to exhibit strong adhesion (Video S1) and to form stable interfacial connections with human skin (Fig. 1c). The coupling between low modulus, high elasticity, and strong adhesion provides RCPTG with excellent mechanical compliance, enabling it to maintain conformal contact and synchronized deformation with the working interface for a long time, which greatly guarantees the working stability of the on-skin flexible sensor devices [36]. By comparing with other reported advanced elastic triboelectric materials, RCPTG has excellent competitiveness, especially in terms of flexibility, triboelectric responsivity, and adhesion properties (Fig. 1d) [37–40]. Functionalized extension of RCPTG using laser etching and mask coating techniques to fabricate RCPTG-skin demonstrated excellent reprocessing performance (Fig. 1e). Combining it with triboelectric sensing technology can mimic the sensing capabilities of human skin, such as tactile sensing and object recognition.

### Phase-Locked Feature Enabled by Competitive Hydrogen Bonding Induced Phase Separation

**Fig. 2:**
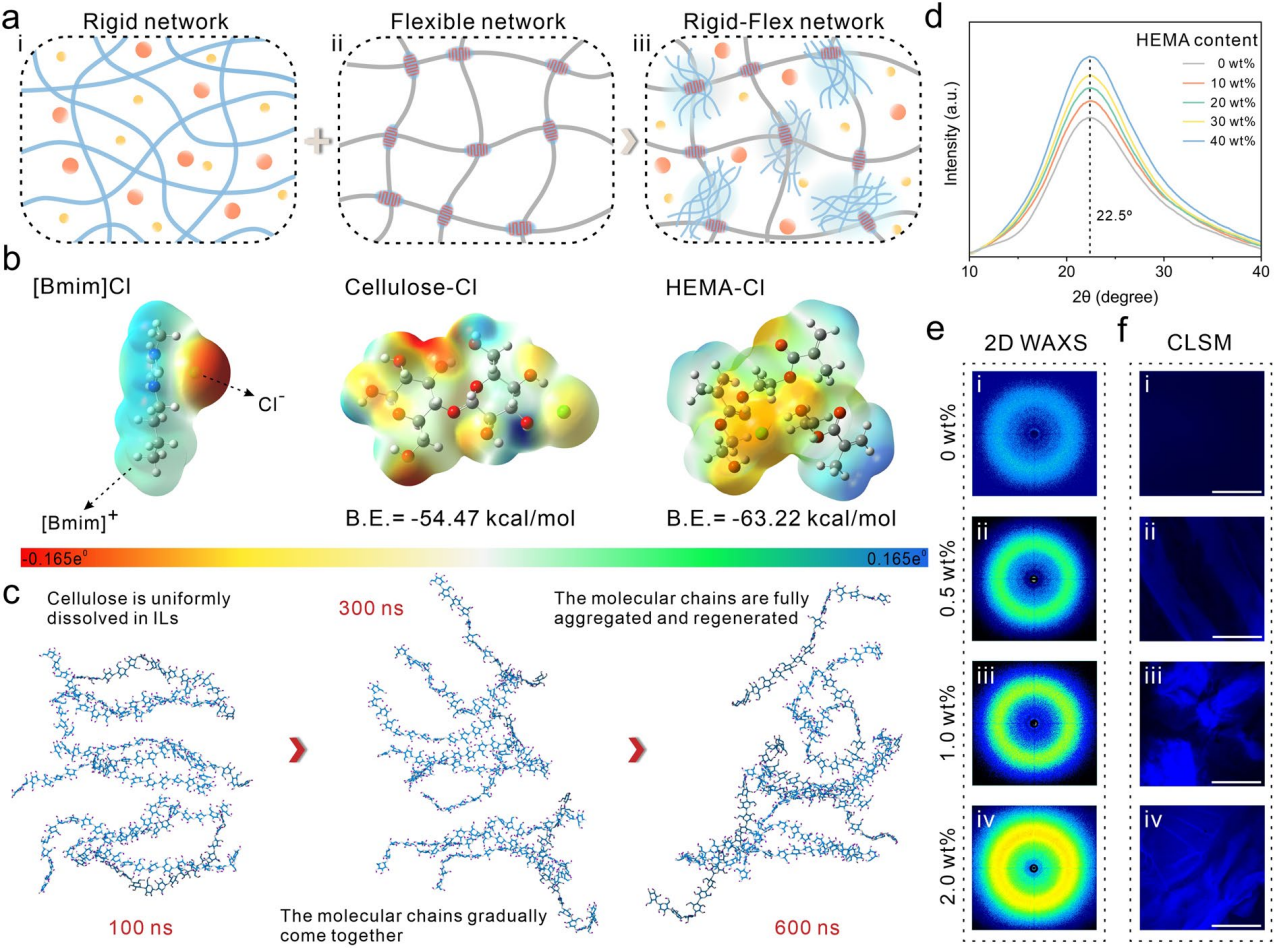
Phase separation induced by hydrogen bonding competition strategy. **a** Schematic diagram of a rigid-flexible network with phase separation features. **b** Process of inducing phase separation through solvent-nonsolvent effects. **c** Snapshots of MD in cellulose regeneration processes. **d** XRD spectra of gels with different HEMA contents. **e** 2D WAXS patterns of gels with different cellulose contents. **f** CLSM images show areas of increasing degree of phase separation, with the bright portion being the RC after staining, scale: 200 μm

Phase separation already occurs in the precursor before the successful preparation of the gel (Video S2). The cellulose network homogeneously dissolved in [Bmim]Cl acts as rigid backbone in the precursor (Fig. 2a-i). HEMA, a nonsolvent for cellulose, triggers the phase separation while acting as polymerization monomer for the flexible network (soft phase) (Fig. 2a-ii). Due to the strong affinity of HEMA, the Cl^−^ used to dissolve cellulose is heavily contested. Cellulose chains that lose Cl^−^ reform interchain hydrogen bonds and regenerate into amorphous RC (hard phase), forming a precursor solution with phase-separation features (Fig. 2a-iii). The regeneration of cellulose was verified using Fourier transform infrared spectroscopy (FTIR), and the -OH stretching vibrational peak at 3345 cm^−1^ shifted in the short-wave direction, demonstrating the formation of intermolecular hydrogen bonds in cellulose (Fig. S1) [41]. XPS results also demonstrated the composition of the chemical bonds within the gel (Fig. S2). Density functional theory (DFT) calculations demonstrated that the driving force for phase separation may originate from two competing hydrogen bonds in the system. The interaction and binding energies of Cellulose-Cl, HEMA-Cl were − 64.46 and − 102.79 kcal mol^−1^, − 54.47 and − 63.22 kcal mol^−1^, respectively (Fig. 2b), indicating that Cl^−^ preferentially forms stronger and more stable hydrogen bonds with HEMA. The phase separation process was further verified using molecular dynamics (MD) simulations (Figs. 2c and S3). With the addition of HEMA, the cellulose molecular chains, which were originally homogeneously dissolved in [Bmim]Cl, were aggregated due to the loss of Cl^−^, and the degree of aggregation further deepened with the reaction time. The stable regeneration of cellulose is further evidenced by the decrease in solvent accessible surface area of the system and the change in the number of hydrogen bonds of each component (Fig. S4).

To verify the promotion of phase separation by the non-solvent, the crystal structure inside the precursor was analyzed by XRD (Fig. 2d). The broad diffraction peak around 22.5° proves that the cellulose regenerated from the ionic liquid is amorphous [42]. The intensity and width of the diffraction peaks increased with the increase in HEMA addition, proving the promotion of cellulose regeneration by HEMA. When the system is free of cellulose, there are no observable scattering rings in the two-dimensional WAXS pattern (Fig. 2e-i), indicating that the system is very homogeneous internally. As the cellulose content increases, brighter scattering rings are observed (Figs. 2e-ii-iv and S5). At the same time, the WAXS spectra also showed progressively enhanced scattering peaks (Fig. S6), indicating that a clear and increasing degree of phase separation was generated within the system [43]. CLSM images (Figs. 2f-i-iv and S7) and fluorescence microscopic images (Fig. S8) can intuitively observe the generation of phase separation.

### Rheology and Environmental Stability of Iontronic Triboelectric Gels with Phase-Locked Structures

**Fig. 3:**
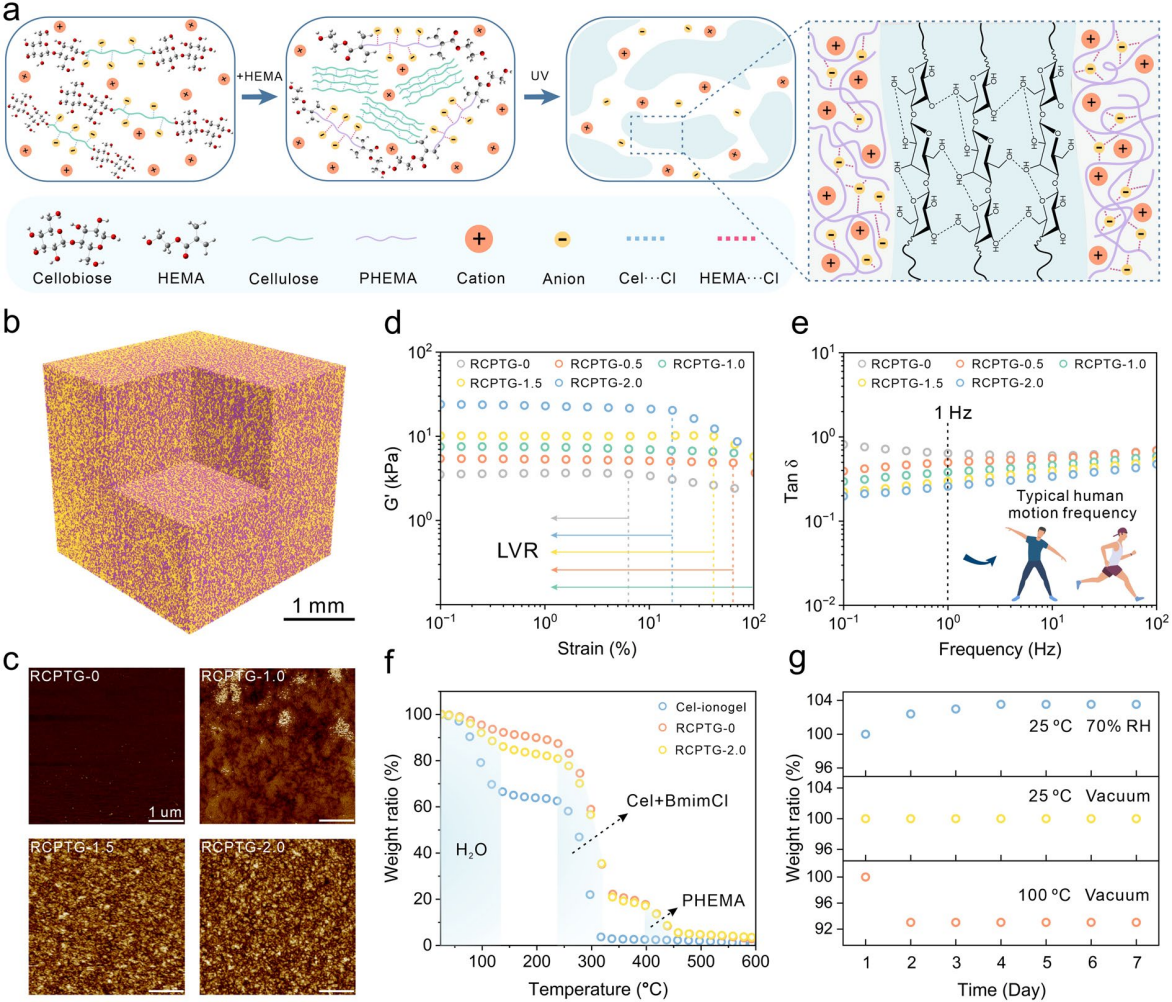
Effect of phase separation on rheological and environmental stability of triboelectric gels. **a** Preparation process of RCPTG with phase-locked structure. **b** Computed tomography reconstruction of phase-locked structures in RCPTG by nano-CT. **c** AFM phase diagram of RCPTGs. **d** The linear viscoelastic region of RCPTGs. **e** Frequency loss factors for RCPTGs. **f** TGA analysis of RCPTGs. **g** Moisture absorption and environmental stability of RCPTGs

To prepare triboelectric gels with phase-locked structure, rigid phases of varying sizes were locked in soft matrix using UV curing (Figs. 3a and S9). The internal structure of the RCPTG can be visualized by Nano Computed Tomography (Nano CT) scanning (Fig. 3b). Similar to traditional phase separation, the amorphous-polymer-dense phase and gelated-polymer-sparse phase form bicontinuous phase-locked structures of micrometer scale [44]. The phase-locked structure in RCPTG was further verified by atomic force microscopy (AFM) (Figs. 3c and S10). The results clearly show the aggregation of soft phase (dark area) and hard phase (bright area). As the system is uncrystallized, the aggregation of hard phases at different scales acts as physical cross-linking and reinforcement [45], which has an impact on the viscoelastic behavior of the gel, especially in terms of stress response and deformation. Rheological studies have shown that the viscosity of RCPTG is positively correlated with the mass fraction of RC (Fig. S11), and excessive regeneration of cellulose will cause the gel to exhibit macroscopic phase separation and no longer maintain morphological homogeneity (Fig. S12). As can be seen from the shear strain scans, the storage modulus (G') of RCPTG is positively correlated with the rigid phase content, which is due to the ability of rigid chains to store more elastic energy (Fig. S13). The linear viscoelastic region (LVR) shows an increasing and then decreasing trend with increasing rigid phase content (Fig. 3d). This may be because a small amount of RC fills the gaps in the flexible network, increasing the continuity and homogeneity within the gel. When cellulose is regenerated in large amounts, the larger-sized rigid phase occupies a more flexible volume, further limiting the linear deformation space [46]. The loss modulus (G'') of the gel is less than G' (Fig. S14) for all tested frequencies, exhibiting a constitutive gel state, which stems from the dynamic reversibility exhibited by its internal network [47]. The loss angle tangent (Tan *δ*) of RCPTGs is less than 1 in all frequency test ranges (Fig. 3e, 1 Hz is the frequency of normal human movement) and temperature ranges (Fig. S15), indicating that they maintain a stable solid-like morphology even under complex frequency and temperature conditions.

To verify the suitability of triboelectric gels in real-world application environments, a series of endurance tests were performed, including thermodynamic stability, hygroscopicity, and ionic liquid leakage. The thermodynamic stability of RCPTG was checked by thermogravimetric analysis (TGA). Compared to the cellulosic ionogel without PHEMA, RCPTG showed lower mass loss at the same temperature, which resulted from less water inside the gel and the high thermodynamic stability of PHEMA (Figs. 3f and S16). The high hygroscopicity and leakage risk of ionic liquids is a pain point issue that has long plagued the stability of ionic gel applications. Here, the weight change of RCPTG under different temperature and humidity environments was checked (Fig. 3g). RCPTG absorbed only about 4 wt% water from the environment over 7 days, which is much lower than the moisture absorption level of the same-component ionogel [48]. No significant ionic liquid leakage was observed within 48 h after the successful gel preparation (Fig. S17), which was attributed to the large number of anions being attracted by the strong hydrogen-bonding donors and being confined in the phase-locked structure [49], which reduced the water molecule binding sites and ensured the stability of the gels in the practical application environment. In addition, the appearance of the gel becomes slightly cloudy with increasing RC content (Fig. S18), which is due to photorefraction caused by deeper phase separation. Notably, excellent compatibility between the components was maintained (Fig. S19), ensuring the molding ability and processing properties of the triboelectric gels. This resulted in RCPTGs maintain more than 80% light transmission in the visible range (Fig. S20), which meets the high demand for material transparency in flexible electronic devices.

### Compliant Mechanical Properties Endowed by Phase-Locked Structure

**Fig. 4:**
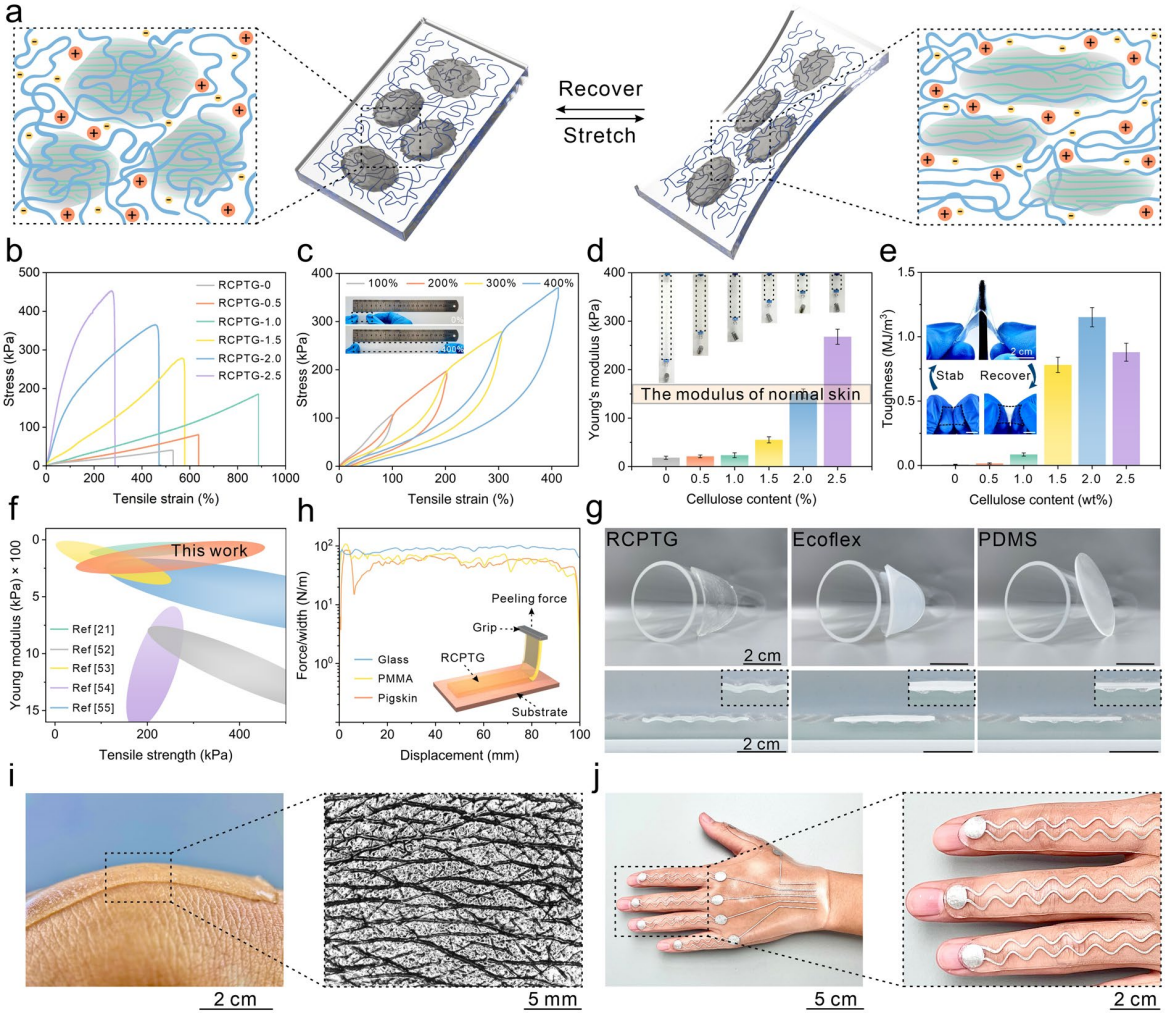
Skin-like compliant mechanical properties enabled by phase separation strategy. **a** Interphase load transfer mechanism in phase-locked structure of RCPTG. **b** Tensile stress–strain curve. **c** Stretching cycle curves, insets show the original state and the RCPTG stretched to 400%, respectively. **d** Young’s modulus of RCPTGs, where the Young’s modulus of RCPTG-2.0 is within the modulus interval of human skin tissue. Inset: RCPTGs suspended with 100 g weights. **e** Comparison of toughness of RCPTGs. Inset: puncture resistance of RCPTG. **f** Comparison of modulus and strength of RCPTG with reported elastic materials. **g** Comparison of RCPTG with commercial gels (Ecoflex, PDMS). **h** Peel strength of RCPTG with glass, PMMA, and pigskin, respectively. **i** RCPTG forms a favorable conformal contact with human epidermal wrinkles. **j** RCPTG-skin with flexible circuitry and its detailed view

The key to obtaining excellent skin-like mechanical properties of RCPTG is an effective interphase load transfer mechanism. Upon stress, the rigid and brittle RC hydrogen-bonded network preferentially breaks and sacrificially dissipates a large amount of energy; the sparsely crosslinked flexible polymer network acts as a hidden length, which is dramatically stretched upon stress and withstands the stress through large deformation (Fig. 4a) [50]. As the phase separation deepens, the fracture stress of the gel increases, while the fracture elongation, as in the case of viscoelastic behavior, shows a tendency to increase and then decrease (Fig. 4b). This is because a large amount of flexible space is occupied when the rigid phase size exceeds the critical value. At the same time, the strong inter-polymer association makes the dense region too rigid to continue to act as a sacrificial bond for energy dissipation [51]. Benefiting from the reversible hydrogen bonding in the rigid phase and the moderate cross-linking of the flexible network in the soft phase, RCPTG did not exhibit significant hysteresis under cyclic loading with gradually increasing strain and no interval (Fig. 4c). Phase separation enhances the RCPTG while increasing Young’s modulus from the unavailable range (18.4 kPa) to a level comparable to that of human skin (~ 100–200 kPa) (Fig. 4d). In terms of toughness, the best toughness of up to 1.15 MJ m^−3^ is about 165 times higher than that of the original sample, demonstrating good puncture resistance (Fig. 4e and Video S3). For compression, the cyclic curve exhibits a closed hysteresis loop, indicating excellent elastic recovery (Fig. S21 and Video S4). The mechanical properties of RCPTG described above, especially the low Young’s modulus and high strength, are highly competitive among the reported skin-like elastic materials (Fig. 4f) [21, 52–55]. It is noteworthy that competitive hydrogen bond-induced phase separation is a versatile toughening strategy for gels. In addition to cellulose, similar results have been achieved in natural polymers such as chitosan, silk protein, and soluble starch (Fig. S22).

Tactile sensing systems are typically set up in non-planar positions, such as joints and non-planar epidermis in the human body, and irregular housings in robots. Therefore, the ability to make a compliant connection with the subject becomes an important indicator for evaluating triboelectric gels. Compared to the commercial elastomers Ecoflex and PDMS, RCPTG not only forms conformal contacts with irregular objects but also forms stable interfacial connections with fingers without additional adhesives (Figs. 4g and S23). The PHEMA in the soft phase has a large number of hydroxyl groups and therefore forms strong hydrogen bonds or electrostatic interactions with most common materials [56], obtaining a peel strength over 70 N m^−1^ (Fig. 4h). At the same time, low cytotoxicity and excellent bacteriostatic properties demonstrate the safety of RCPTG for application on human skin (Figs. S24 and S25). BmimCl can achieve antimicrobial effects at low concentrations, and the ion-binding effect conferred by the phase-locked structure prevents it from causing significant irritation to human tissues [57]. It also does not cause itching or inflammation of the skin when in contact with the human body for a long period (Fig. S26). When adhered to the human body for a long time, RCPTG can even completely replicate the microtexture of the epidermis (Fig. 4i). Conductive silver paste can be introduced to the gel surface using a mask coating technique to form a stretchable and flexible electrode layer that together form a triboelectric tactile skin (RCPTG-skin) (Fig. 4j). It can maintain stable and compliant contact with the human body (Video S5), which lays a promising foundation for the advanced fabrication of skin-like soft electronics.

### Wearable Self-Powered Tactile Sensing based on Compliant Iontronic Triboelectric Gels

**Fig. 5:**
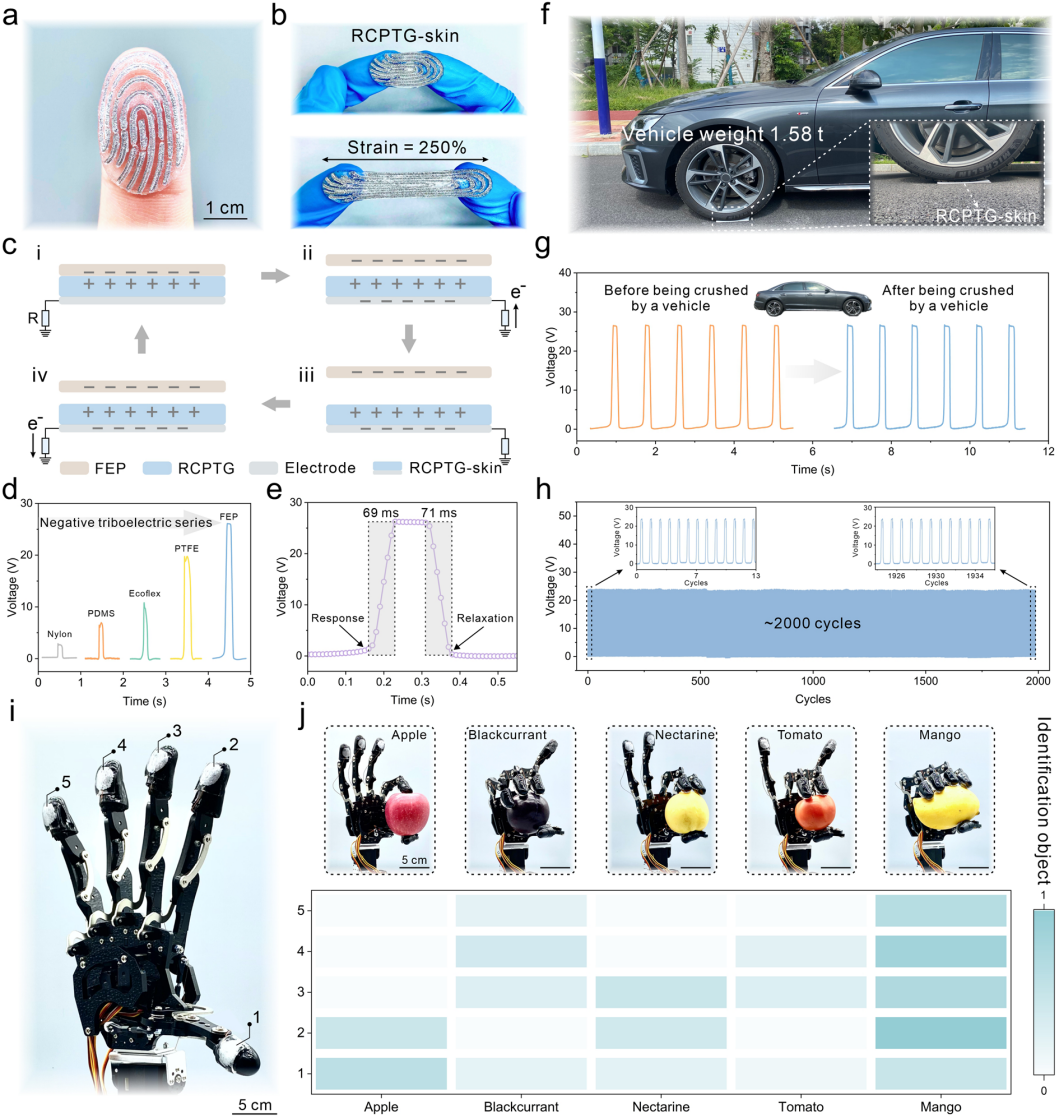
Self-powered perception properties of triboelectric tactile skin. **a** Compliant RCPTG-skin configured on a finger. **b** Images of RCPTG-skin before (top) and after (bottom) being stretched. **c** Self-powered sensing mechanism of RCPTG-skin based single-electrode triboelectric nanogenerator. **d** Contact electrification properties of RCPTG-skin paired with different commercial materials. **e** Self-powered response and relaxation time of RCPTG-skin. **f** Image of RCPTG-skin when subjected to destructive crushing by a vehicle weighing 1.58 t. **g** Comparison of the triboelectric output of RCPTG-skin before and after crush. **h** Sensing stability of RCPTG-skin at ~ 2000 cycles. **i** Image of a triboelectric tactile sensory system. **j** A robotic hand integrated with self-powered tactile skin serves as an operable haptic gripper that recognizes grasping motions and strengths based on the magnitude of triboelectric signals

The RCPTG-skin was assembled into a self-powered prosthetic skin that was used to mimic the tactile sensory ability of human skin (Fig. 5a). Due to the high stretchability and flexibility of triboelectric gels, RCPTG-skin can withstand 250% strain without failure (Fig. 5b). Based on RCPTG-skin, a stretchable single-electrode triboelectric nanogenerator was composed of RCPTG and conductive silver paste as triboelectric layer and electrode layer, respectively. Self-powered tactile signal sensing was realized through the coupling effect of contact electrification and electrostatic induction (Figs. 5c-i-iv and S27). The triboelectric sensing mechanism gives the RCPTG-skin sensing properties that mimic the tactile sensation of a finger, enabling the material recognition properties of the skin. When a finger loaded with RCPTG-skin comes into contact with an object of different polarity, the electrical output signal increases with the elevation of the negative triboelectric series (Fig. 5d). This is due to the abundant hydroxyl dipoles on RC and PHEMA that make RCPTG display a higher triboelectric positive polarity, and the amount of transferred charge increases with the widening of the polarity gap of the triboelectric materials [58]. When fully contacted with a common triboelectric negative polarity material, fluorinated ethylene propylene copolymer, RCPTG-skin produced open-circuit voltages, short-circuit currents, and transferred charges of 37 V, 1.2 μA, and 9.5 nC, respectively (Fig. S28). While possessing strong triboelectric properties, the RCPTG-skin exhibits fast response and relaxation speeds. The response and relaxation times are 69 and 71 ms (Fig. 5e), respectively, and such fast responsivity is competitive even with rigid materials that do not produce elastic deformation [59].

Pressure sensing sensitivity is particularly important for evaluating the tactile sensing performance of elastic triboelectric materials. The sensitivity of the triboelectric signal of the RCPTG-skin was 0.383 kPa^−1^ when the pressure was less than 4.2 and 0.013 kPa^−1^ when the pressure was in the range of 4.2–100 kPa, respectively (Fig. S29). The fast and sensitive pressure response capability guarantees the efficiency of the RCPTG-skin in practical tactile sensing applications. In some special application scenarios, especially in the field of robotics or prosthetics, soft tactile sensing devices need to have the ability to withstand transient high stress [60]. The phase-locked structure, which mimics real skin, allows the RCPTG-skin to deform sufficiently reversibly to dissipate the large pressure and recover its initial state when subjected to transient high forces. RCPTG-skin retains its morphological integrity even after being subjected to a transient weight of 1.58 t (Figs. 5f and S30). The triboelectric signals of the RCPTG-skin also did not decay significantly after being crushed by a vehicle (Fig. 5g), indicating that the gel structure was preserved. After approximately 2000 cycles of operation, the triboelectric output performance of the RCPTG-skin did not decay significantly, demonstrating its sensing stability (Fig. 5h). A triboelectric haptic sensing system was further designed (Fig. 5i). When grasping objects made of different materials, the tactile gripper loaded with RCPTG-skin feeds back distinctly differentiated triboelectric signals, which will help the robot to achieve accurate object recognition (Fig. S31) [61, 62]. By normalizing the triboelectric signals, a thermogram of the electrical signals corresponding to the grasped object is obtained (Fig. 5j). Since the triboelectric signal is proportional to the contact pressure, the grasping gesture and force can be recognized from the thermogram, enabling the robot to better perceive and process the tactile signals. The high mechanical robustness and sensitive object sensing capability provide the possibility of further applications of RCPTG-skin in the fields of rescue and search, military training, and deep space exploration.

## Conclusions

A regulatory mechanism is proposed in this study, based on competitive hydrogen bonding that induces phase-locked structures, leading to the successful customization of triboelectric gels with biomimetic-compliant mechanical characteristics. Leveraging the dissolution-regeneration process of natural polymers, non-solvents are introduced to establish a competitive hydrogen bonding system, initiating liquid–solid phase separation. Natural polymers devoid of hydrogen bonds spontaneously regenerate, forming the rigid phase for toughness, while flexible polymer networks with hydrogen bonds constitute the soft phase to maintain viscoelasticity. The phase-locked structure with alternating soft-hard phases enables the gel to maintain skin-like softness, characterized by Young’s modulus of 150.6 kPa; while significantly increasing mechanical strength, resulting in an 810% improvement in strength and 1650% enhancement in toughness. The resulting triboelectric tactile skin, which is built upon this gel, establishes a stable, compliant interface with the human body, showcasing an excellent combination of self-powered sensing mechanisms and biomimetic characteristics. This study rationally utilizes the phase separation phenomenon induced by hydrogen bond competition, opening doors to skin-like compliant designs of triboelectric gels, with the potential to advance the field of bio-sensors, including soft robotics, electronic skin, and tactile sensors.

## Supplementary Information

Below is the link to the electronic supplementary material.

Supplementary file1 (MP4 1947 kb)

Supplementary file2 (MP4 9107 kb)

Supplementary file3 (MP4 3293 kb)

Supplementary file4 (MP4 3718 kb)

Supplementary file5 (MP4 4660 kb)

Supplementary file6 (DOCX 4890 kb)
