# Supplementary material for: Compliant Iontronic Triboelectric Gels with Phase-Locked Structure Enabled by Competitive Hydrogen Bonding
Source: Nanomicro Lett. 2024 Apr 9;16:170. doi: 10.1007/s40820-024-01387-4 (PMC11003937; doi:10.1007/s40820-024-01387-4)
Supplement: Supplementary file 6 — Supplementary file6 (DOCX 4890 kb) [file 40820_2024_1387_MOESM6_ESM.docx]

Supporting Information for

**Compliant Iontronic Triboelectric Gels with Phase-Locked Structure Enabled by Competitive Hydrogen Bonding**

Guoli Du^1^, Yuzheng Shao^1^, Bin Luo^1^, Tao Liu^1^, Jiamin Zhao^1^, Ying Qin^1^, Jinlong Wang^1^, Song Zhang^1^, Mingchao Chi^1^, Cong Gao^1^, Yanhua Liu^1^, Chenchen Cai^1^, Shuangfei Wang^1^, Shuangxi Nie^1^*

^1^School of Light Industry and Food Engineering, Guangxi University, Nanning 530004, P. R. China

*Corresponding author. E-mail: [nieshuangxi@gxu.edu.cn](mailto:nieshuangxi@gxu.edu.cn) (Shuangxi Nie)

**S1 Experimental Section**

**S1.1 Density Functional Theory (DFT) Calculation**

The structures of cellobiose, HEMA, and their complexes with Cl^-^ were fully optimized at the B3LYP-D3BJ/def2-SVP level of theory. The vibrational frequencies of the optimized structures were carried out at the same level. The structures were characterized as a local energy minimum on the potential energy surface by verifying that all the vibrational frequencies were real. The interaction energy (ΔE) of the optimized cluster was calculated. ΔE is defined as the difference between the complex and the sum of energies of monomers, which can be obtained by the following formulas ΔE =E(AB)-E(A)-E(B). Both the molecular electrostatic potential (MEP) and the interaction energy were realized by Gaussian 16.

**S1.2 Molecular Dynamics (MD) Simulation**

MD simulations of cellulose system was performed using GROMACS 2021.5 package. Cellulose, which consists of 16 β-D-glucoses, was parameterized by GLYCAM-06j-1 force field. [Bmim]^+^ and HEMA were geometrically optimized by Gaussian 16 under density functional theory B3LYP/def-TZVP level with DFT-D3 dispersion correction. The general AMBER force field 2 (GAFF2) parameters were used. Initial cubic box with a size of 11.5×11.5×11.5 nm^3^ was established by extending at least 1.4 nm outward along pre-built 10 chains of cellulose and the system was solvated in [Bmim]Cl and HEMA ([Bmim]Cl: HEMA=1:1), then and energy minimization was performed by using the steepest descent algorithm with a force tolerance of 500 kJ mol^-1^ nm^-1^. In all three directions, periodic boundary conditions were imposed. Then these systems were relaxed for 1 ns under NPT ensemble and position restraints with a constant of 1000 kJ mol^-1^ nm^-2^ in three directions were performed on heavy atoms of celluloses and cubic box size eventually stabilized at 9.3×9.3×9.3 nm^3^. After completing the above steps, 600 ns NPT MD simulations with a timestep of 2 fs were performed. The system pressure was controlled at 1 bar and the temperature was controlled at 363K. Lennard-Jones interactions were calculated within a cutoff of 1.2 nm, and electrostatic interactions beyond 1.2 nm were treated with the particle-mesh Ewald (PME) method with a grid spacing of 0.16 nm.

**S2 Supplementary Figures**


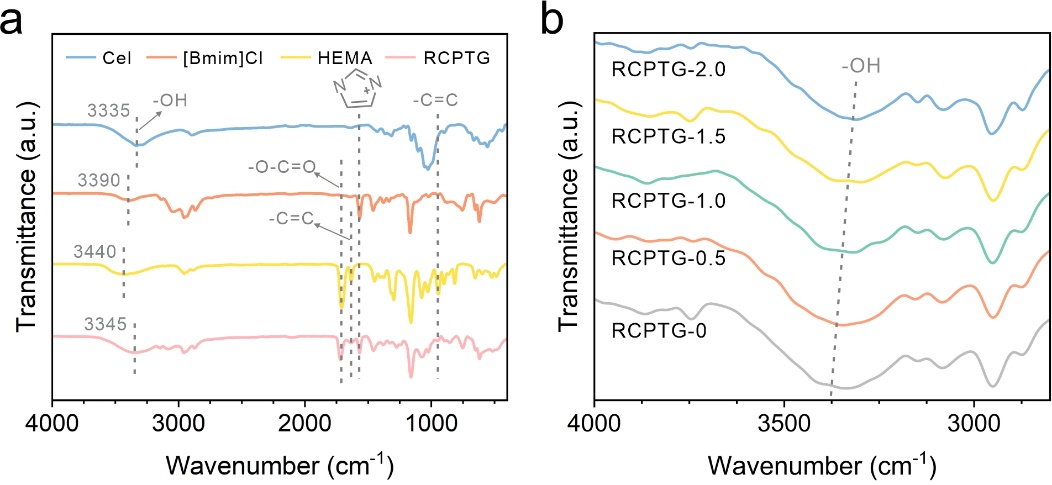


Fig. S1 FTIR analysis of RCPTG. a FTIR spectra of RCPTG and its components, including cellulose, [Bmim]Cl, and HEMA. b FTIR spectra of RCPTG with different cellulose contents


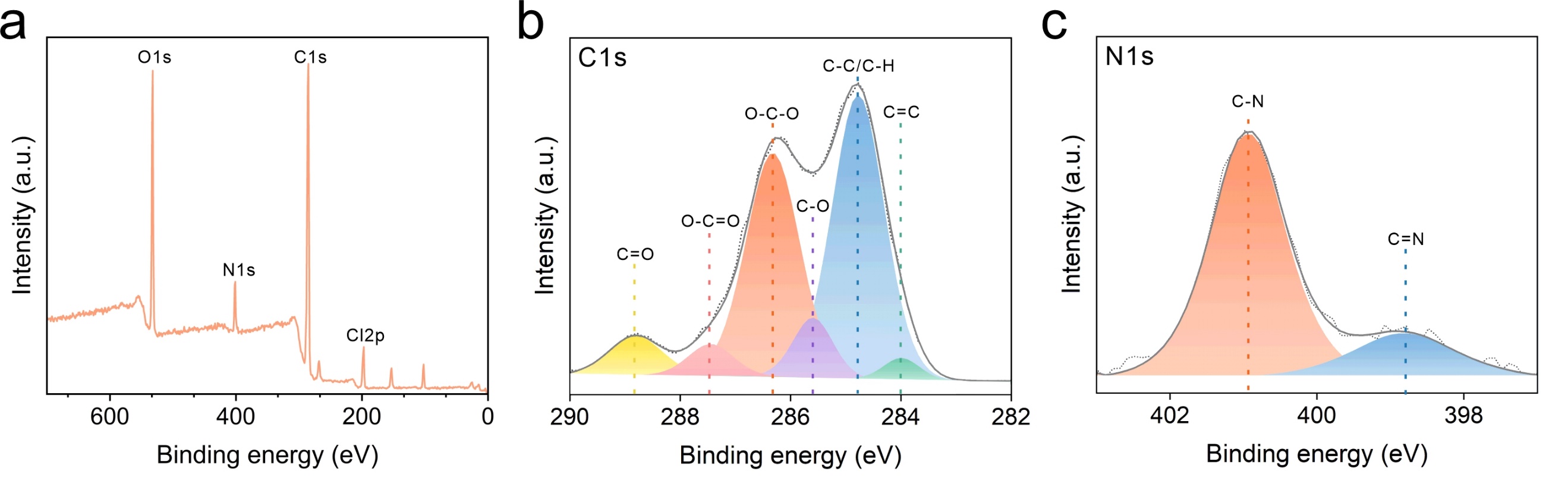


Fig. S2 XPS analysis of RCPTG. a XPS full elemental spectrum of RCPTG. b C 1s spectra. c N1s spectra

RCPTG and its component raw materials are analyzed by FTIR and the results are shown in Fig. S1a. In RCPTG, the characteristic peak at 3345 cm^-1^ is assigned to -OH in regenerated cellulose; the characteristic peak at 1718 cm^-1^ is attributed to -O-C=O in HEMA, and the effective polymerization of HEMA is also evidenced by the significant decrease of the -C=C telescoping peak at 1630 cm^-1^; and the characteristic peak at 1568 cm^-1^ is attributed to the imidazole ring in [Bmim]^+^. The -OH characteristic peaks shifted towards the short-wave direction indicating the creation of more intramolecular hydrogen bonds (Fig. S1b), proving a substantial regeneration of cellulose. The chemical structure of the elastomer is analyzed using high-resolution XPS spectroscopy, and typical N-featured peaks and Cl-featured peaks are found in RCPIE (Fig. S2a). The O-C=O bonding in PHEMA, and C-N, C=N bonding in ionic liquids are evidenced by C 1s and N 1s spectroscopy, respectively (Fig. S2b, c)


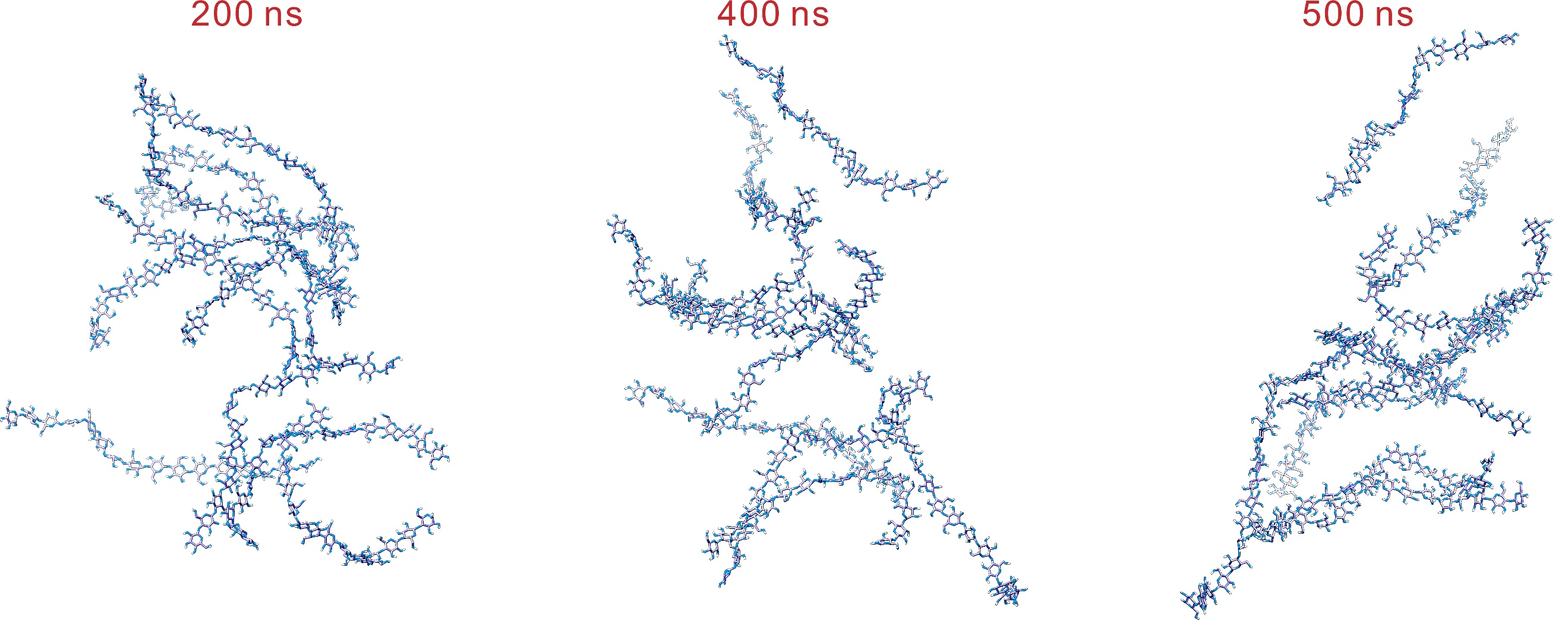


Fig. S3 MD snapshots at 200, 400, and 500 ns


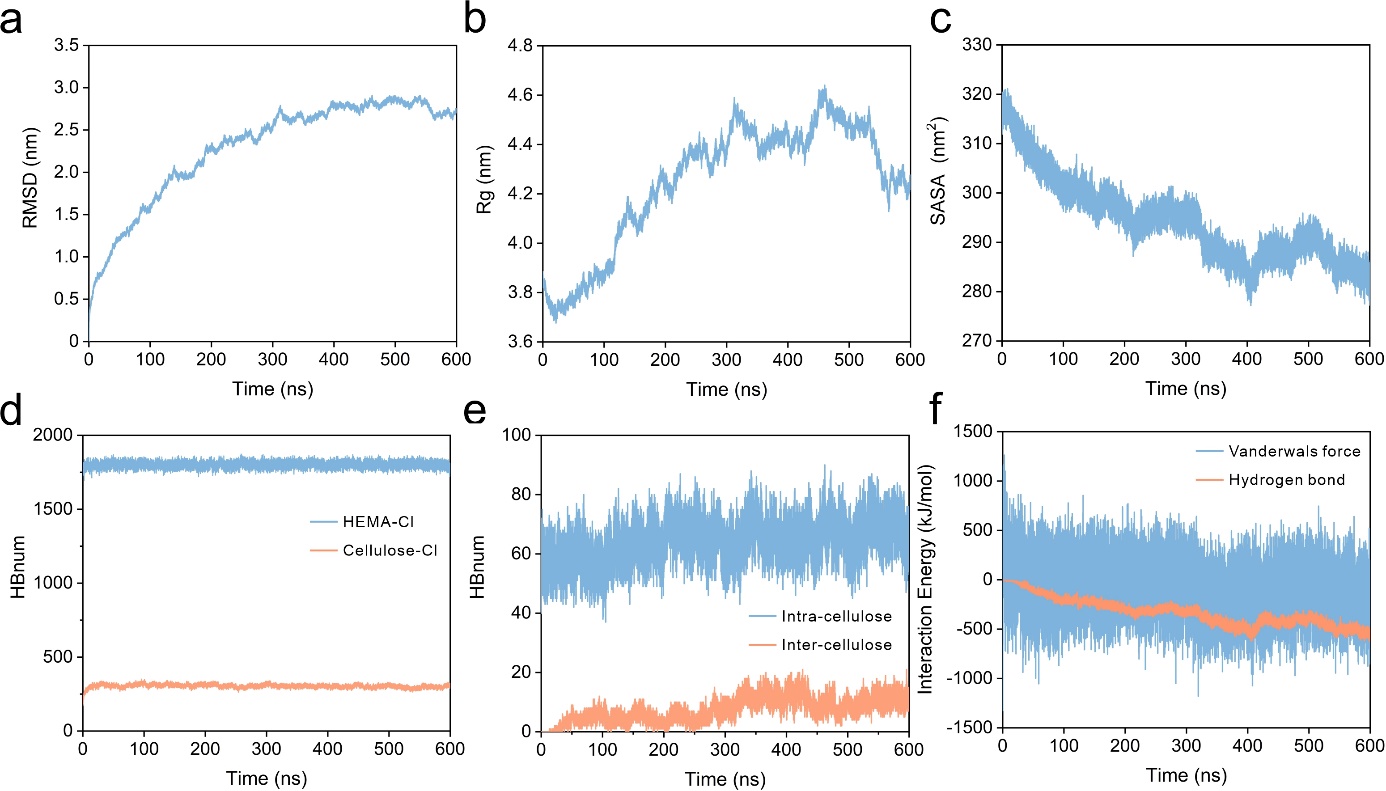


Fig. S4 MD simulation results. a RMSD of cellulose molecular chains with simulation time. b Rg of cellulose molecular chains with simulation time. c SASA of cellulose molecular chains with simulation time. d Variation of the number of two competing hydrogen bonds (HEMA-Cl and Cellulose-Cl) with simulation time. e Variation of the number of intramolecular and intermolecular hydrogen bonds in cellulose with simulation time. f Cellulose chain interaction energy with simulation time

MD simulations of the hard-phase generation process in RCPTG are performed. HEMA is added as nonsolvent to the homogeneously solubilized cellulose/[Bmim]Cl system to construct a competitive hydrogen-bonding system (HEMA-Cl, Cellulose-Cl). With the addition of HEMA, significant aggregation of the originally dispersed cellulose molecular chains occurred, regenerating amorphous cellulose. The root mean square deviation (RMSD) is used to measure the difference between the polymer structure and the initial conformation in each frame of the MD simulation process, and is mainly used as an important basis for determining whether the simulated system has reached equilibrium. The radius of gyration (Rg) is used to measure the degree of tightness of the macromolecule, which is also an important equilibrium indicator of the simulated system. As the simulation proceeds, especially in the last 50 ns, both RMSD and Rg tend to stabilize (Fig. S4a, b), indicating that the conformation of regenerated cellulose tends to stabilize. In addition, the solvent-accessible surface area (SASA) in the system also demonstrated the regeneration of cellulose (Fig. S4c). The SASA showed a decreasing trend with increasing HEMA. This is due to the effective aggregation of cellulose chains reducing the area in contact with the solvent. Meanwhile, the strong anionic attraction of HEMA-Cl is evidenced by the change in the number of competing hydrogen bonds in Fig. S4d. The number of intermolecular and intramolecular hydrogen bonds of cellulose likewise proves the regeneration of cellulose (Fig. S4e), i.e., the generation of hard phase in phase separation. Finally, the interaction energy between cellulose chains was calculated (Fig. S4f). The results showed that the van der Waals interaction energy, hydrogen bonding interaction energy and total interaction energy between cellulose chains were -187.74, -517.17, and -704.91 kJ mol^-1^, respectively, which proved the key role of hydrogen bonding in inducing the phase separation process.


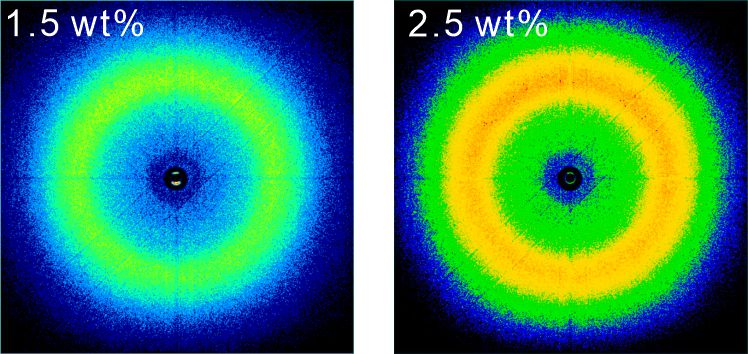


Fig. S5 2D WAXS (supplement to Fig. 2e)


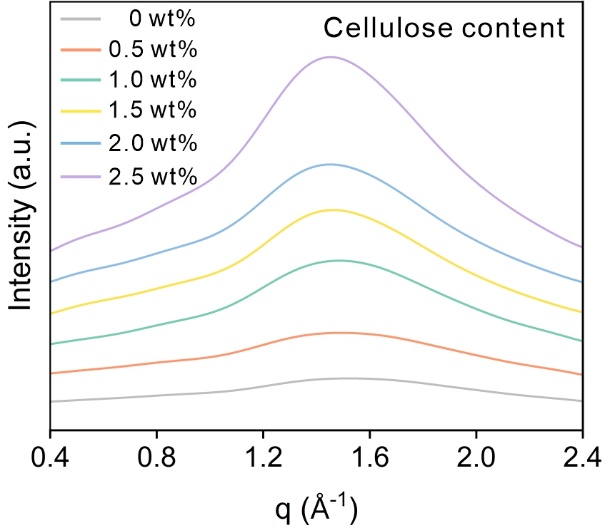


Fig. S6 WAXS spectra of gels with different cellulose contents


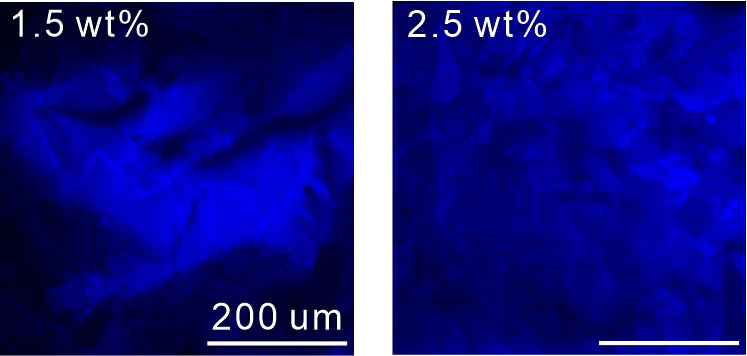


Fig. S7 CLSM image (supplement to Fig. 2f)


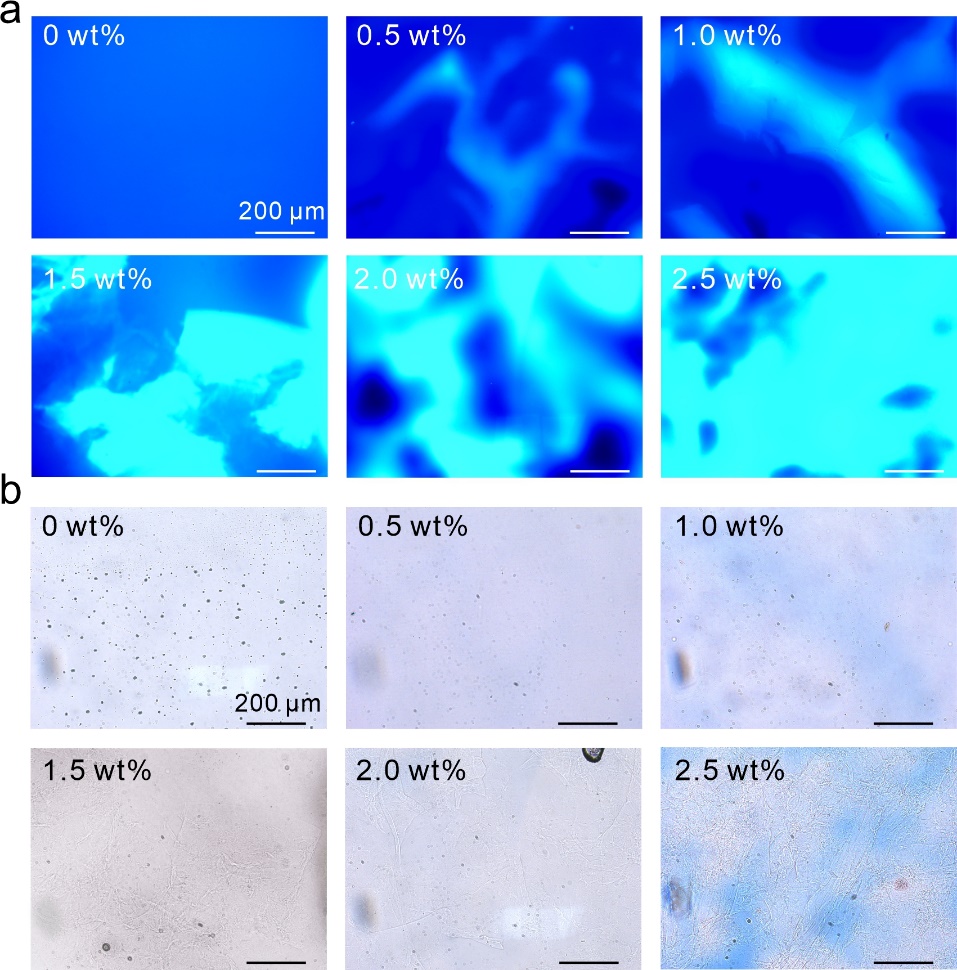


Fig. S8 Fluorescence microscopy images of gels with different cellulose contents. a Fluorescence excitation states. b Non-excited states


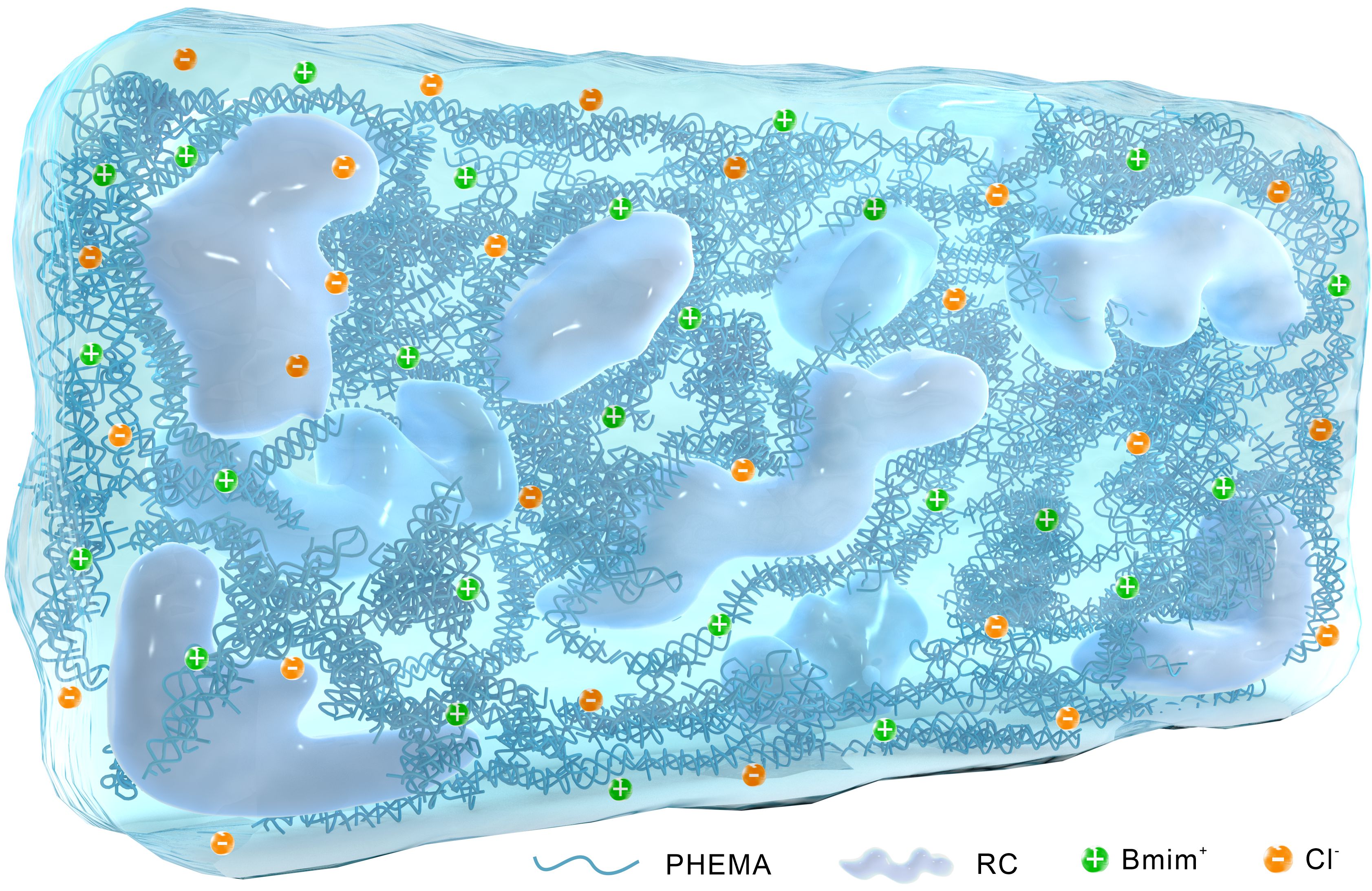


Fig. S9 Schematic diagram of the internal phase-locked structure of RCPTG


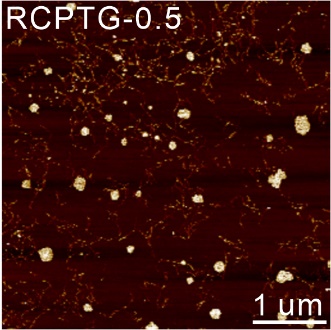


Fig. S10 AFM phase diagram of RCPTG-0.5 (supplement to Fig. 3c)


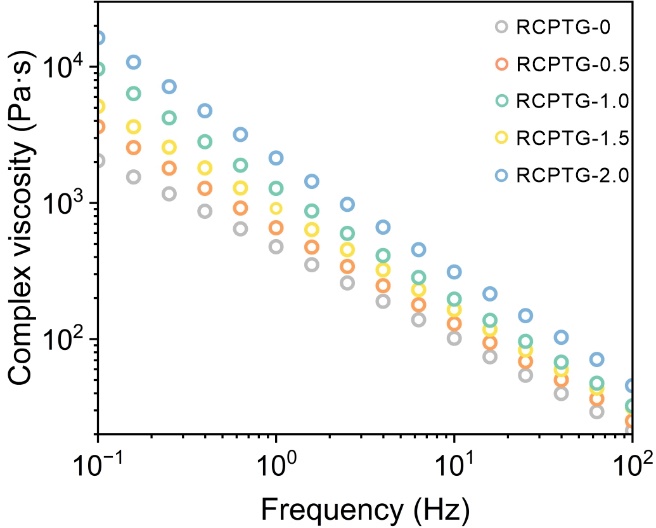


Fig. S11 The complex viscosity of RCPTG


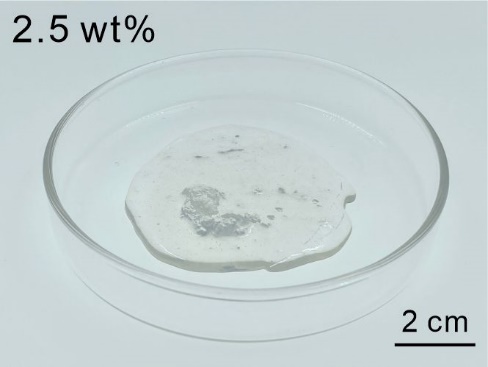


Fig. S12 Triboelectric gel precursor solution containing 2.5 wt% cellulose, with large amounts of cellulose regeneration leading to macroscopic phase separation


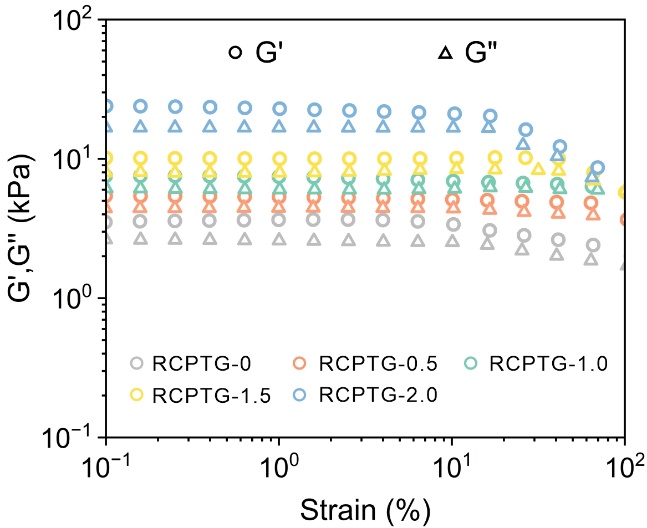


Fig. S13 Shear strain sweep of RCPTGs


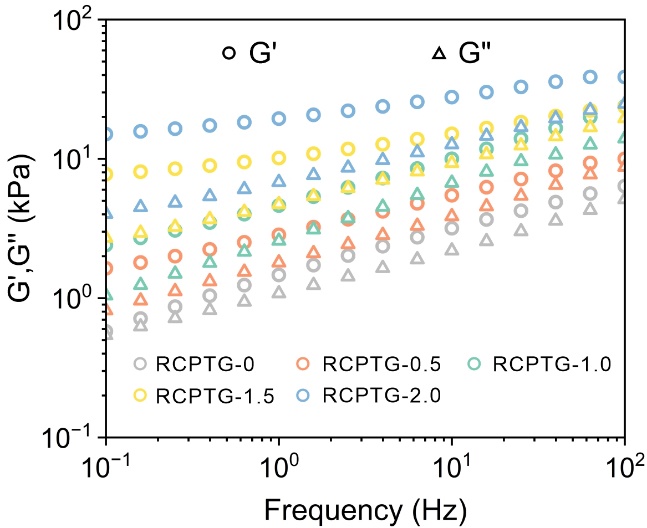


Fig. S14 Frequency sweep of RCPTGs


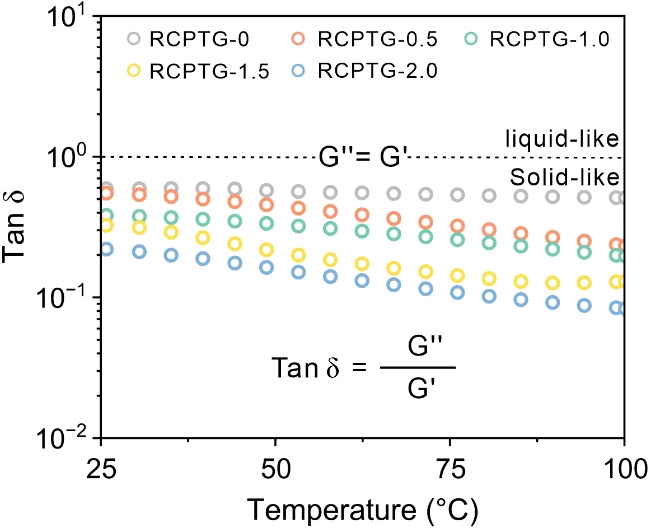


Fig. S15 Temperature-dependent loss factors for RCPTGs


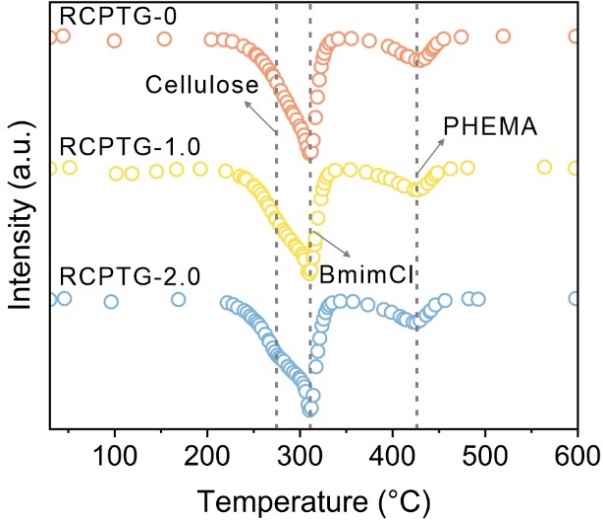


Fig. S16 Derivative thermogravimetric (DTG) analysis of RCPTGs


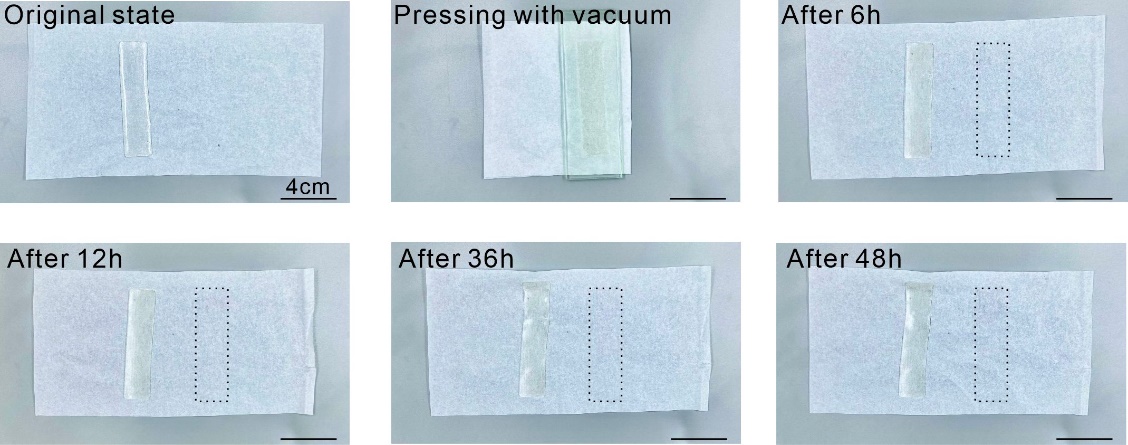


Fig. S17 Inspection of ionic liquid leakage in RCPTG. The RCPTG was wrapped in a dry, dust-free paper towel pressed firmly with a heavy object, and placed in a vacuum environment for observation of ionic liquid leakage. Within 48 h, no traces of liquid infiltration were seen on the paper towel, proving the good anti-leakage performance of RCPTG


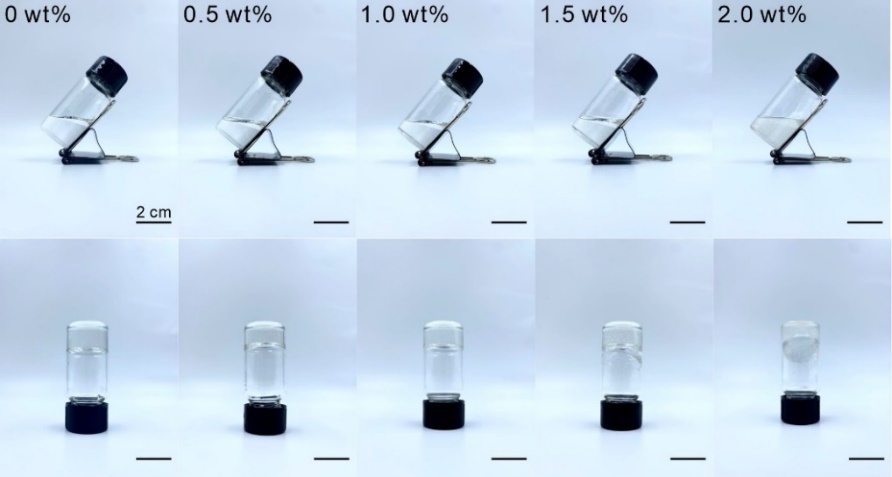


Fig. S18 The precursor solution before curing (top row), and the gels after curing (bottom row)


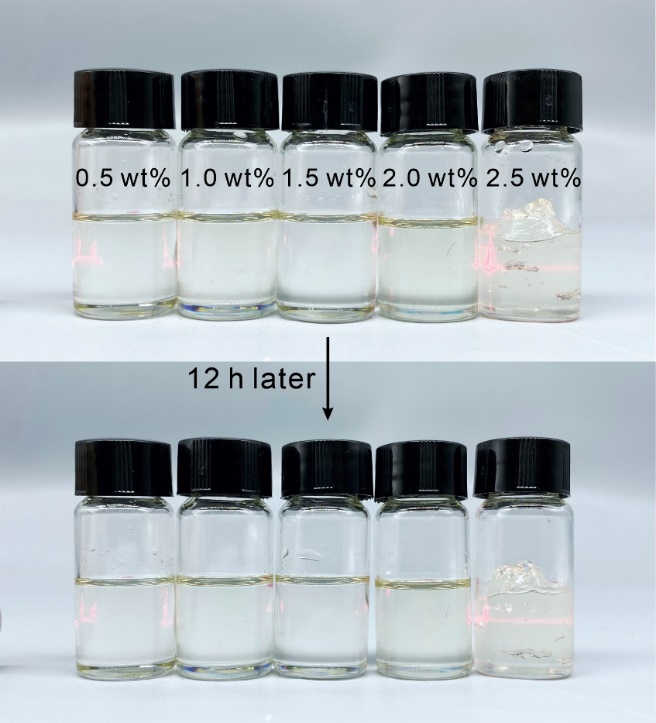


Fig. S19 Compatibility validation of cellulose/BmimCl/HEMA system at different cellulose contents


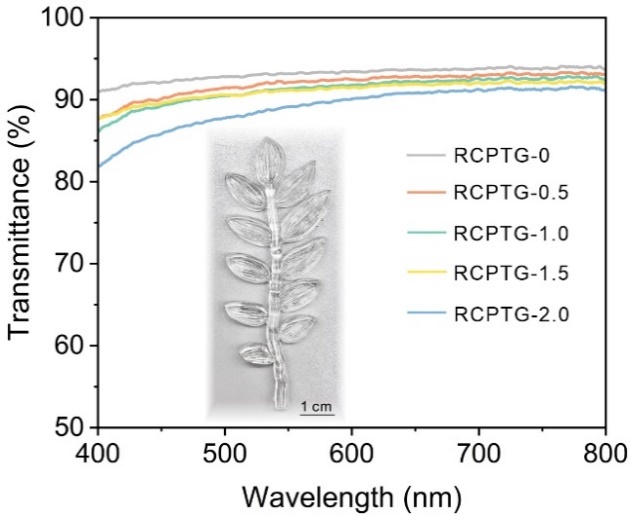


Fig. S20 Transmittance of RCPTGs in the visible wavelength range. Inset: highly transparent leaf model with fine leaf texture made by RCPTG-2.0 via inverted mold


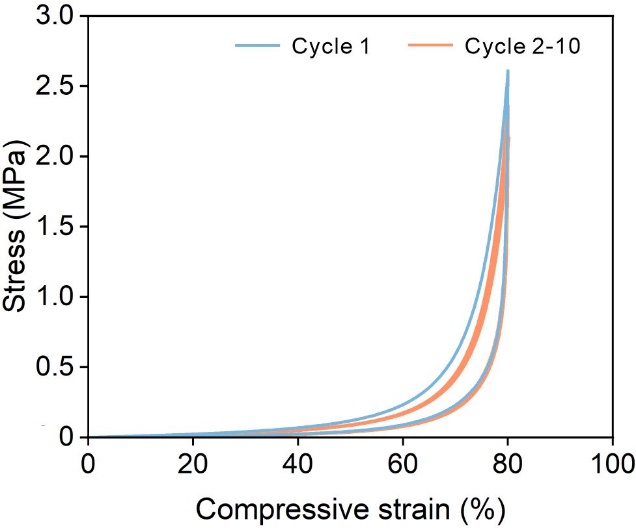


Fig. S21 Compression cycle curves for RCPTG-2.0. There is no significant hysteresis in any of the cycles except the first one


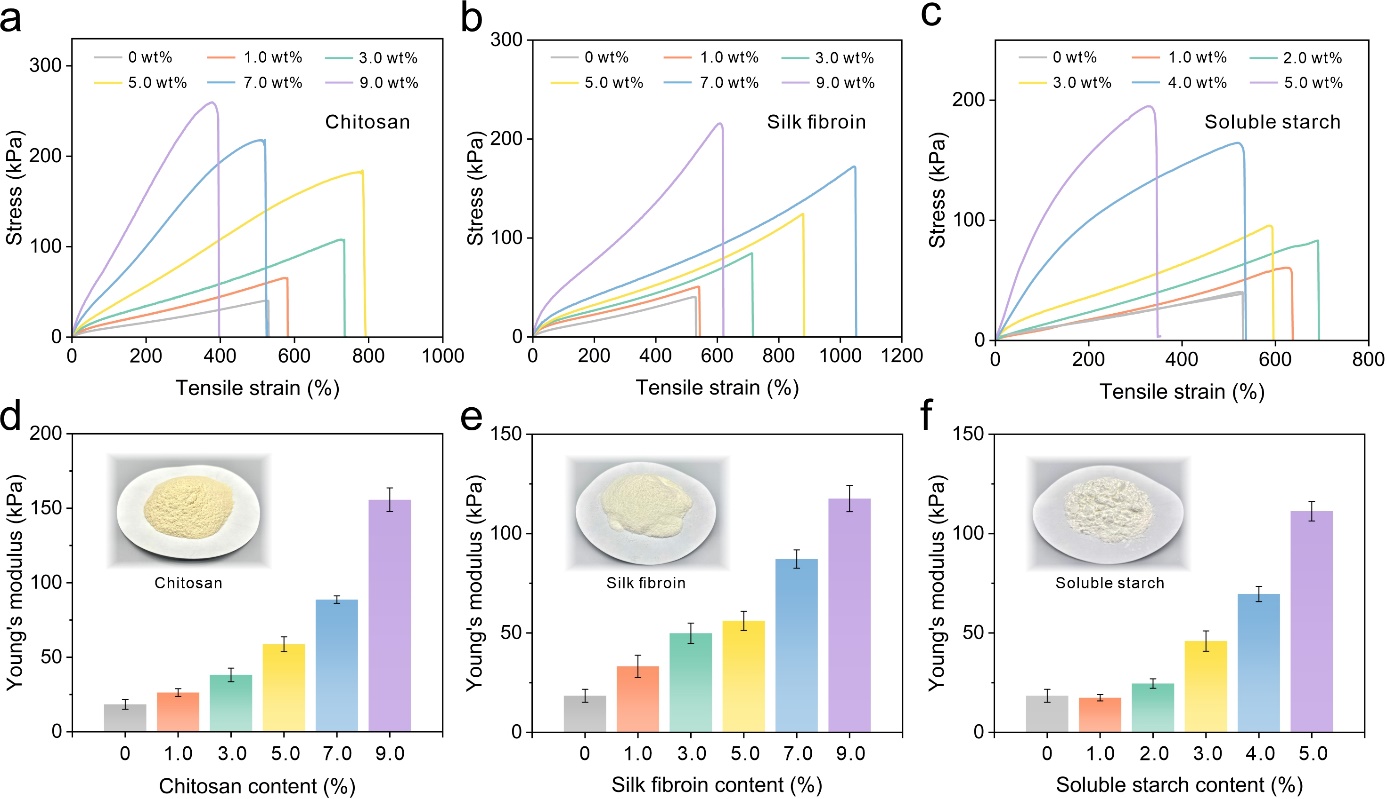


Fig. S22 A generalized strategy for reinforcing gels by competitive hydrogen bonding-induced phase separation. Replacement of cellulose with chitosan, silk protein, and soluble starch gives the same strengthening trend. a-c Tensile stress-strain curve. d-f Variation of Young's modulus. The inset shows an optical picture of the biomass raw materials


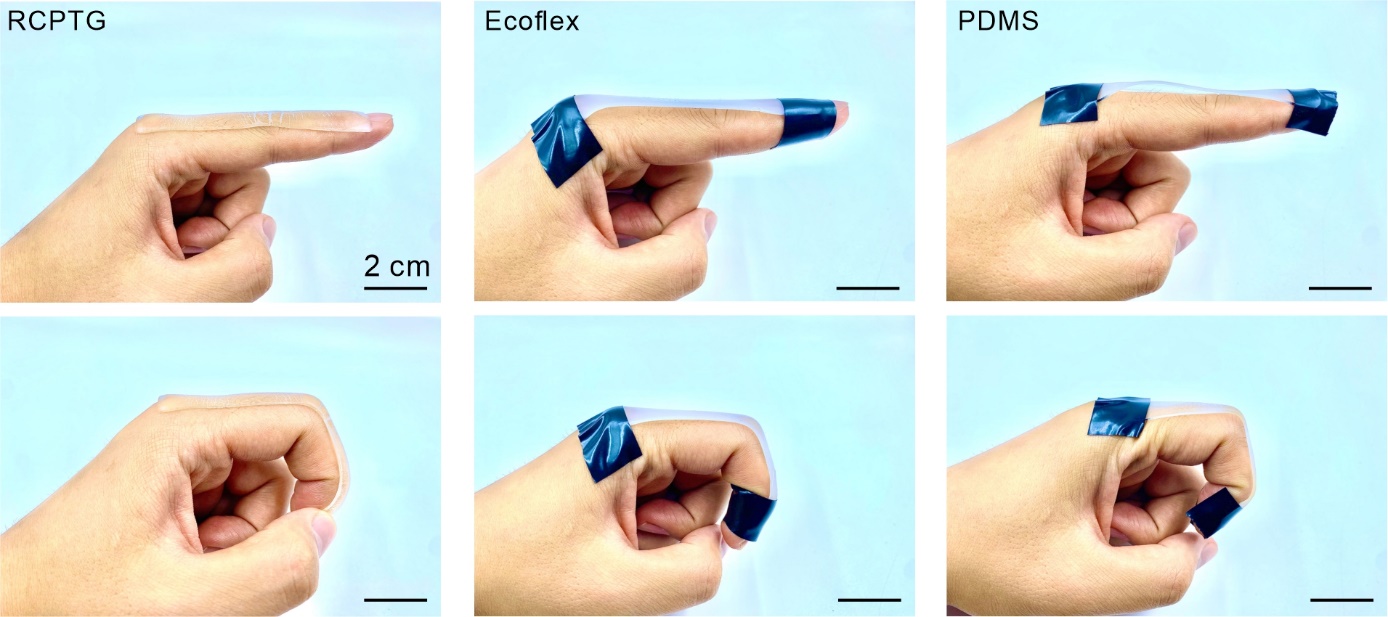


Fig. S23 Comparison of RCPTG and commercial gels (Ecoflex, PDMS) regarding human compliance


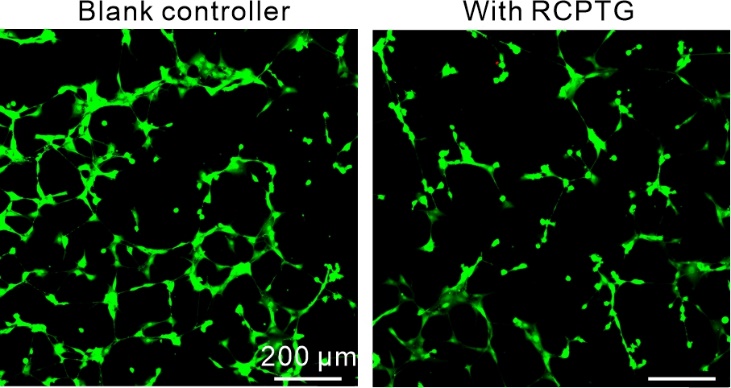


Fig. S24 Cytotoxicity testing for RCPTG. Co-culturing RCPTG with NIH3T3 cells for 48 h did not result in significant cell death


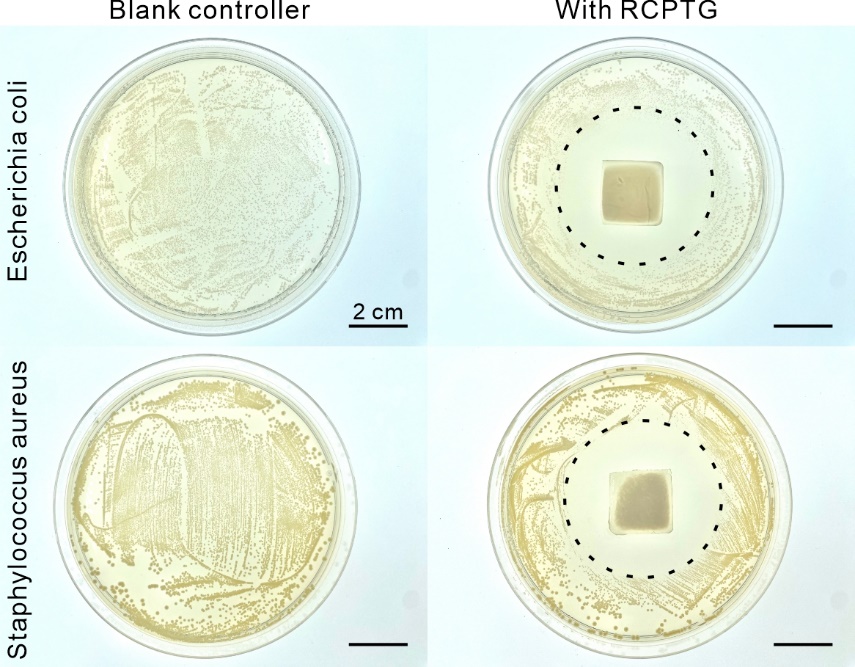


Fig. S25 Bacteriostatic performance testing for RCPTG. A significant inhibition zone appeared in the bacterial medium co-cultured with RCPTG


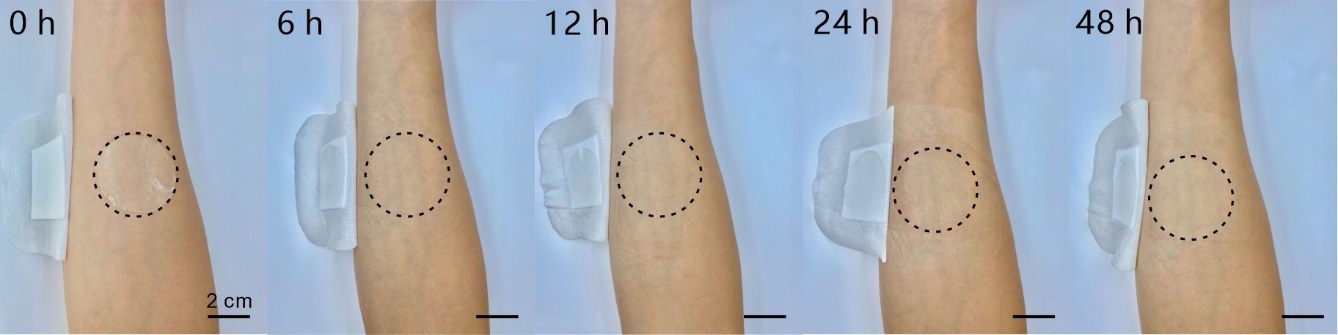


Fig. S26 Compatibility testing of RCPTG on human skin


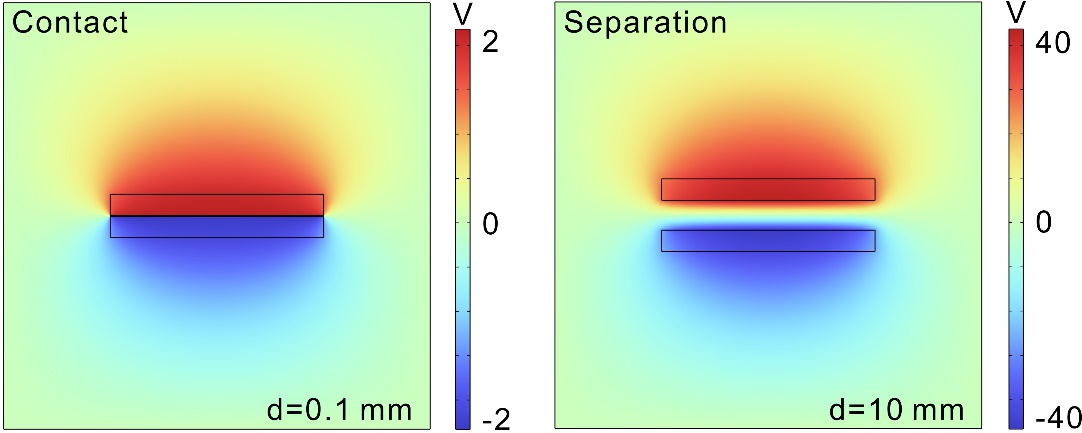


Fig. S27 COMSOL simulation of the working process of RCPTG-based single electrode triboelectric nanogenerator


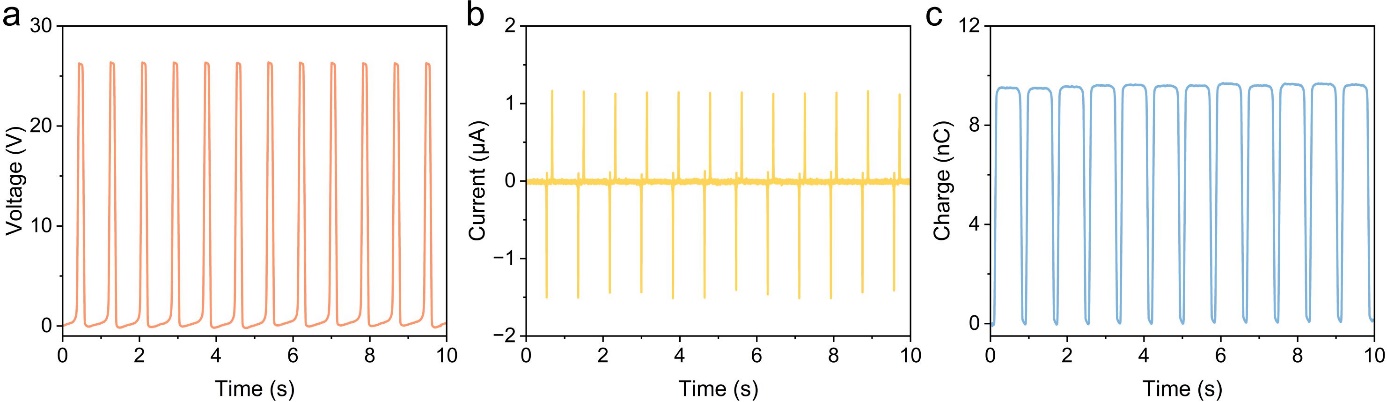


Fig. S28 Triboelectric output properties of RCPTG-skin (pair with FEP). a Open-circuit voltage. b Short-circuit current. c Transfer charge


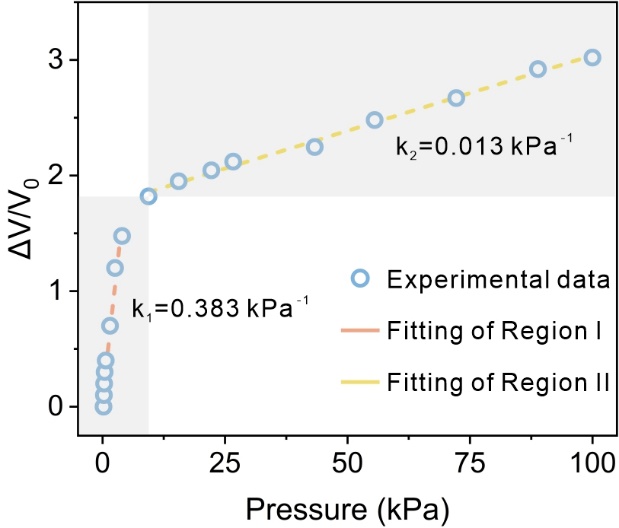


Fig. S29 Pressure sensing sensitivity for RCPTG tactile skin


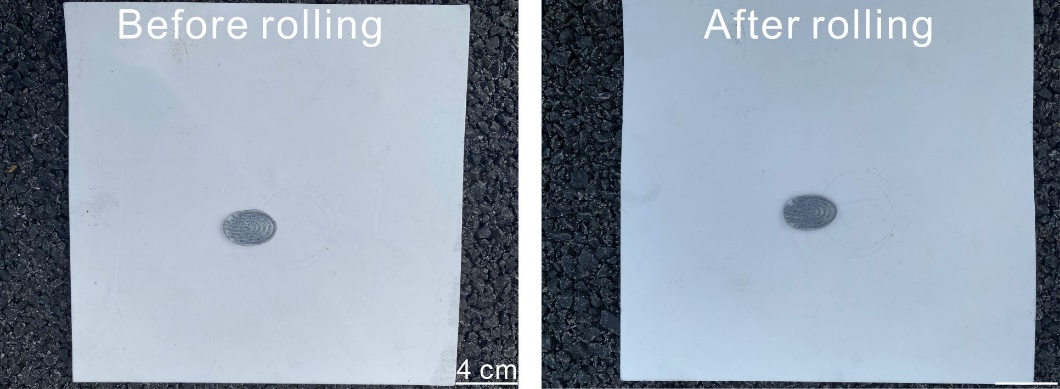


Fig. S30 Comparison of RCPTG morphology before and after vehicle rolling


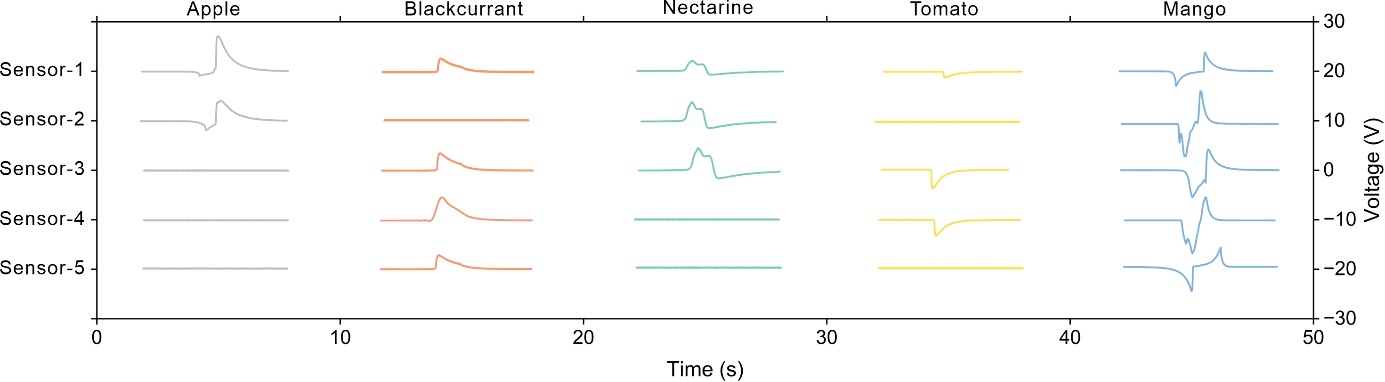


Fig. S31 Utilizing the electrical signals generated by a triboelectric manipulator after gripping different objects. The difference between the peak shape and the value of the voltage is used to recognize the gripping posture and strength of the manipulator
